# Triplex DNA clamp regulates Cas12a activation for ssDNA and RNA sensing

**DOI:** 10.1093/nar/gkaf1392

**Published:** 2026-01-08

**Authors:** Andrea Celeste Di Pede, Neda Bagheri, Erica Belforte, Alessio Palone, Marianna Rossetti, Alessandro Porchetta

**Affiliations:** Department of Chemical Science and Technologies, University of Rome, Tor Vergata, Via della Ricerca Scientifica 1, Rome 00133, Italy; Department of Chemical Science and Technologies, University of Rome, Tor Vergata, Via della Ricerca Scientifica 1, Rome 00133, Italy; Department of Chemical Science and Technologies, University of Rome, Tor Vergata, Via della Ricerca Scientifica 1, Rome 00133, Italy; Department of Chemical Science and Technologies, University of Rome, Tor Vergata, Via della Ricerca Scientifica 1, Rome 00133, Italy; Department of Chemical Science and Technologies, University of Rome, Tor Vergata, Via della Ricerca Scientifica 1, Rome 00133, Italy; Department of Chemical Science and Technologies, University of Rome, Tor Vergata, Via della Ricerca Scientifica 1, Rome 00133, Italy

## Abstract

We present a molecular strategy that enables the programmable activation of the CRISPR–Cas12a system in response to triplex DNA formation triggered by single-stranded DNA (ssDNA) or RNA inputs. Our triplex-controlled Cas12a assay leverages the high specificity of clamp-like triplex structures to control a toehold-based strand displacement reaction within a rationally designed DNA hairpin (PAM-Switch). Upon displacement and protospacer adjacent motif (PAM) complementation, the Cas12a ribonucleoprotein (RNP) is activated, initiating *trans*-cleavage and producing a concentration-dependent fluorescent signal. By decoupling target recognition (via triplex formation) from direct hybridization with the Cas12a–crRNA complex, the assay eliminates the need for target-specific crRNAs. This design also allows multiple detection of distinct nucleic acid (NA) targets using a single Cas12a reaction mix. Through the use of triplex-based clamps, the proposed platform achieves enhanced specificity for single-nucleotide variants and supports the detection of both ssDNA and RNA targets across a broad range of lengths (10–20 nucleotides), addressing key limitations in current Cas12a-based diagnostics and opening new avenues for NA sensing.

## Graphical Abstract

**Graphical Abstract:**
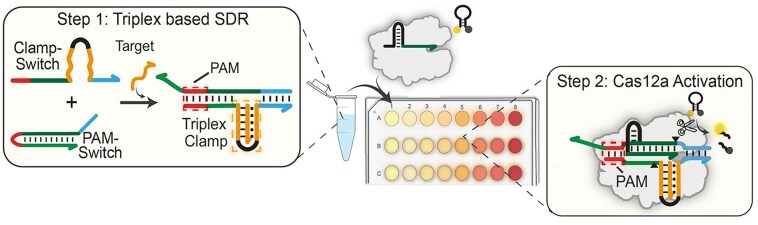

## Introduction

Clustered regularly interspaced short palindromic repeats (CRISPR)-based toolbox aims to create the next generation of *in vitro* molecular diagnostics for routine clinical use. Since the discovery of the collateral (i.e. *trans*-) cleavage activity of RNA and single-stranded DNA (ssDNA) by CRISPR–Cas type V and VI systems, a variety of biomolecular sensors and assays have been reported for molecular diagnostics [1–6]. Among them, platforms based on the RNA-programmed Cas12 effector have gained particular interest as Cas12 contains a single RuvC nuclease domain that cleaves complementary double-stranded DNA (dsDNA) adjacent to a T-rich protospacer adjacent motif (PAM) and ssDNA nonspecifically (*trans*-cleavage) [7, 8]. Despite significant advancements toward real-world applications, Cas12a-based molecular assays still face limitations. First, *trans*-cleavage is activated by base-pairing of a nucleic acid (NA) target to a complementary crRNA guide [9]. This requirement imposes the use of a specific ribonucleoprotein (RNP) complex for each DNA target, thus preventing easy multiplexing and increasing the cost of screening analysis. Second, its natural dsDNA-targeting activity has limited its application for RNA detection [10–12]. In this respect, a split-activator highly accessible RNA analysis (SAHARA) strategy has recently been reported, using short RNA inputs, showing RNA substrates activating *trans*-cleavage activity of Cas12a [13]. However, the approach still requires the hybridization between a starter DNA and a crRNA guide (e.g. at least 16 base pairs) [13]. Third, Cas12a systems tolerate some mismatches between the crRNA and the target DNA to prevent immune evasion [14], and so the specificity varies depending on the position and number of mismatches (MM) [15, 16]. This leads to limited detection specificity for single-base mutations [1, 15–17], posing challenges for the identification of disease-related point mutations [18]. In particular, the specificity of the Cas12a detection system is limited to PAM-proximal nucleotides for dsDNA targets, whereas it is not able to detect single base mismatches in ssDNA targets [19]. Therefore, developing molecular strategies to enhance CRISPR specificity is essential to expanding its applicability.

To enhance specificity and better transduce chemical information into biochemical functions, nature often employs binding-induced conformational change mechanisms [20–22]. In particular, clamp-like structure switching mechanism using two recognition elements to embrace a single copy of the target molecule results in enhanced binding affinity with superior specificity for biomolecule detection [23–26]. As an example, molecular chaperones employ clamp-like binding sites to protect unstable protein conformers (due to the larger recognition interface) and guide their transition to the native state [27]. Ring-shaped DNA sliding clamps also take advantage of a clamp-like binding mechanism to control DNA replication and genome maintenance [28]. These observations have inspired the design of clamp-like switches for artificial biotechnologies in the areas of biomolecular sensing and imaging [29–33]. Among them, synthetic clamp-like DNA probes have been used to achieve precise control over specific functions in response to external inputs, especially when designed to be controlled by allosteric effectors [34–37]. A simple approach for engineering a clamp-like DNA-binding mechanism is through triplex DNA, which integrates Hoogsteen interactions with Watson–Crick–Franklin (WCF) base pairing [30]. Triplex switches can be designed to undergo conformational changes that enable the binding of NA targets and the formation of a parallel triple-helix [38–42]. Specifically, the noncanonical DNA triplex structure consists of a polypurine/polypyrimidine duplex, with a third strand binding the major groove via Hoogsteen pairing and aligning parallel to the purine-rich strand. In this configuration, thymine pairs with A-T (TAT triplets), while protonated cytosine pairs with G-C (CG-C^+^ triplets) through Hoogsteen hydrogen bonds [43–45]. The triplex clamp structure offers key advantages, including pH-responsive formation due to Hoogsteen interactions [46, 47]. Additionally, the two-step clamp-like triplex folding improves target specificity and binding affinity [23, 25, 26, 30], yielding enhanced target discrimination compared to duplex-based detection systems [30, 48]. These features have led to the application of clamp-like DNA triplexes in gene regulation [46, 47], nanomedicine [49, 50], and smart drug delivery systems [51, 52].

Building on these findings, we present here a triplex-regulated strategy to precisely control Cas12a activity for molecular sensing applications. Our assay leverages the high specificity of clamp-like DNA triplex for DNA/RNA target recognition in combination with a PAM-engineered DNA hairpin (PAM-Switch) to activate Cas12a *trans*-cleavage [53]. Central to this design is a triplex-controlled toehold-mediated strand displacement reaction (SDR), which induces a hairpin-to-duplex reconfiguration of the PAM-Switch. This mechanism effectively decouples target recognition (via triplex formation) from hybridization with the Cas12a RNP complex [54]. By employing a clamp-like binding mechanism, our system enhances specificity for single-base mutation detection and enables the detection of both ssDNA and RNA targets across a wide range of lengths (from 10 to 20 nt). In addition, it supports multiple detection of distinct NA targets within a single CRISPR reaction mix.

## Materials and methods

### Reagents and materials

Trizma hydrochloride (Tris–HCl), sodium chloride (NaCl), magnesium chloride (MgCl_2_), boric acid (H_3_BO_3_), and diethyl pyrocarbonate (DEPC)-treated water were used as purchased from Merck (St Louis, Missouri). EnGen^®^ Lba Cas12a from *Lachnospiraceae* bacterium ND2006 was purchased from New England Biolabs Inc. (Ipswich, UK). Bst 3.0 DNA polymerase, Nt.BstNBI, dNTPs, and 1× Isothermal Amplification Buffer II were obtained from New England Biolabs (USA).

### Oligonucleotides

Oligonucleotides (HPLC purified) employed in this work were purchased from Metabion International AG (Planegg, Germany) and Biomers.net GmbH (Söflinger, Germany). All sequences were designed using NUPACK or IDT OligoAnalyzer tools and are reported in the Supplementary Information document (Supplementary Tables S1–S5). DNA and RNA oligonucleotides were dissolved in 100 mM Tris–HCl pH 7.5, and DEPC-treated water, respectively to 100 μM concentration. All oligonucleotides were aliquoted and stored at −20 °C until use. To measure the oligonucleotide concentration the Tecan Infinite M200pro (Männedorf, Switzerland) was used with a NanoQuant Plate. A thermocycler was used to anneal oligonucleotides prior to use. Specifically, RNA and DNA oligonucleotides were incubated for 5 min at 65 °C and 2 min at 90 °C, respectively, before being gradually cooled to room temperature (1°C/min) on the benchtop.

### Fluorescence experiments

#### Strand displacement reactions

Fluorescence kinetics experiments were performed using a Cary Eclipse Fluorimeter in 50 µl quartz cuvettes in buffer solution (10 mM Tris–HCl, 50 mM NaCl, and 10 mM MgCl_2_ at pH 7.0) by exciting at ʎ_exc_ = 488 nm and collecting the emission at ʎ_em_ = 520 nm, with 10 nm bandwidth for excitation and 5 nm bandwidth for emission in all experiments. For further details on the specific concentrations of the experimental components, please refer to the figure captions.

#### Triplex-based CRISPR–Cas12a collateral cleavage assay

Experiments were performed by adding the target strand (as detailed in Supplementary Table S3) to a solution containing PAM-Switch (0.5 nM), the corresponding Clamp-Switch (20 nM), and FRET-based DNA reporter (100 nM). The solution was incubated at 37°C for 15 min before transferring an aliquot of 22.5 µl of the sample to a 384-well Tecan black plate, where 2.5 µl of RNP complex (final concentration in solution of 20 nM) was added. Fluorescence intensity was subsequently monitored over time. For specificity assays, the same procedure was followed, but a single concentration of Target 14 nt (5 nM) was tested, and the fluorescence signal was collected after 15 min from the addition. All the experiments were conducted in a buffer containing 10 mM Tris–HCl, 50 mM NaCl, and 10 mM MgCl_2_, pH 7.0 unless otherwise stated.

#### Triplex-based CRISPR–Cas12a collateral cleavage assay for RNA detection

Fluorescence measurements were performed using a Cary Eclipse Fluorimeter in 50 µl of quartz cuvettes. RNA targets (sequences listed in Supplementary Table S3) were added to a solution containing 0.5 nM PAM-Switch, 20 nM Clamp-Switch RNA (CS_RNA), and 100 nM FRET-based DNA reporter, and incubated at 37 °C for 15 min. Fluorescence was then monitored following the addition of 5 µl of pre-assembled RNP complex (final concentration: 20 nM; pre-incubated at 37°C for 30 min at 200 nM). All experiments were conducted in 10 mM Tris–HCl, 50 mM NaCl, and 10 mM MgCl_2_, pH 7.0, unless otherwise specified. Kinetics experiments were conducted using an excitation wavelength fixed at ʎ_exc_ = 488 nm and acquisition at ʎ_em_ = 520 nm, using 5 nm bandwidth in excitation and 5 nm bandwidth emission in all the experiments.

#### Triplex Clamp-based multiplexing array

Fluorescent experiments were conducted using the same experimental procedure previously described in “*Triplex-based CRISPR-Cas12a collateral cleavage assay*” More specifically, here, we added one fixed concentration of one target strand (5 nM) or a combination of them (Target 14 nt, Target 14 nt _V2, and Target 14 nt _V3, as detailed in Supplementary Table S3) to a solution containing one single specific Clamp-Switch (CS_ Target 14 nt, CS_ Target 14 nt _V2, or CS_ Target 14 nt _V3, as listed in Supplementary Table S2) and the same PAM-Switch (0.5 nM). Afterward, an aliquot of 22.5 µl from each sample was transferred to a 384-well Tecan black plate, and 2.5 µl of the same Cas12a reaction mix (20 nM crRNA–Cas12a RNP complex and 100 nM DNA reporter) was added. Data reported refer to the fluorescence signal collected after 15 min from the addition of the DNA target.

#### Triplex Clamp-Switch CRISPR–Cas12a assay with NEAA pre-amplification step

Each Nicking Enzyme-Assisted Amplification (NEAA) reaction (25 μl of total volume) was performed using 2.5 μl of DNA template, 100 nM of forward and reverse primers, 320 μM dNTPs, 10 U Nt.BstNBI, 4.8 U Bst 3.0 DNA polymerase, 1× Isothermal Amplification Buffer II, and 1.8 mM Mg²⁺ (including the Mg²⁺ supplied by the buffer), in 25 mM Tris–HCl (pH 7.9), 50 mM NaCl, and 50 μg/ml bovine serum albumin (BSA). Prior to the NEAA reaction, Nt.BstNBI, Bst 3.0 DNA polymerase, Isothermal Amplification Buffer II, and buffer solution were mixed to prepare Solution A, while NEAA primers, dNTPs, and template DNA were combined to prepare Solution B. The two solutions were incubated separately at 57.6 °C for 1 min, then briefly centrifuged and mixed. The combined solution (NEAA reaction mixture) was subsequently incubated at 57.6 °C for 10 min to allow isothermal amplification. The reaction was then heated to 98 °C for 10 min to inactivate the enzymes and stop the reaction. Following amplification, 7.5 μl of the reaction mixture was transferred into 75 μl of a detection solution containing PAM-Switch (0.5 nM), Clamp-Switch (CS_Target 20 nt, 20 nM), and a FRET-based DNA reporter (100 nM), and incubated at 37 °C for 15 min. Finally, 22.5 μl of the resulting mixture was transferred to a black 384-well Tecan microplate, 2.5 μl of RNP (20 nM) was added, and fluorescence intensity was continuously monitored over time.

### Data analysis

The signal gain (%) is calculated from fluorescence intensity collected after 15 min from the addition of the specific target unless otherwise stated. It represents the fluorescence signal change due to the collateral cleavage activity of Cas12a calculated using the following formula:


\begin{eqnarray*}
\textit{Signal}\ \textit{Gain}\ \left( \% \right) = \frac{{F - {{F}_0}}}{{{{F}_0}}}x\ 100
\end{eqnarray*}

where *F* is the fluorescence generated by the addition of the DNA/RNA input, and *F*_0_ is the fluorescence background of the solution containing all the components (PAM-Switch probe, Clamp-Switch and the CRISPR reaction mix as previously described) in the absence of the input, respectively.

Relative Fluorescence Units (RFU) represent the fluorescence intensity calculated by subtracting the background fluorescence, i.e. the fluorescence signal of the reaction mixture (PAM-Switch probe, Clamp-Switch, and CRISPR reaction mix) in the absence of the target, from the fluorescence measured in the presence of the target:


\begin{eqnarray*}
RFU = F - {{F}_0}
\end{eqnarray*}

where $F$ is the fluorescence signal measured upon addition of the specific target, and ${{F}_0}$ is the fluorescence of the system without the target.

Fluorescence intensity was further normalized using the following formula:


\begin{eqnarray*}
\textit{Norm}.\ \textit{Fluo}.\ = \frac{{\left( {{{F}_t} - {{F}_{min}}} \right)}}{{\left( {{{F}_{max}} - {{F}_{min}}} \right)}}
\end{eqnarray*}

where ${{F}_t}$ is the background-corrected fluorescence at time *t*, ${{F}_{min}}$ is the minimum fluorescence observed across all samples, and ${{F}_{max}}$ is the maximum fluorescence measured across all concentrations. This allows direct comparison of both kinetic profiles and relative signal intensities.

Binding curves were fitted with the following equation:


\begin{eqnarray*}
y = {{y}_0} + \frac{{{{{\left[ {\textit{Target}} \right]}}^{{{n}_H}}}\left( {{{y}_S} - {{y}_0}} \right)}}{{{{{\left[ {\textit{Target}} \right]}}^{{{n}_H}}} + {{K}_{1/2}}^{{{n}_H}}}}
\end{eqnarray*}

Where ${{y}_{}}\ $is the fluorescence signal in the presence of different target concentration; ${{y}_0}$ is the fluorescence in the absence of target; ${{y}_S}$ is the fluorescence in the presence of a saturating concentrations of target; ${{K}_{1/2}}$ is the concentration of the target at a half-maximum; $[ {\textit{Target}} ]$ is the concentration of the target and ${{n}_H}\ $is the Hill coefficient.

The limit of detection (LOD) was calculated as the ratio of three times of the standard deviation of the blank to the slope of the linear regression. All these analyses were completed using GraphPad Prism 8.0. Error bars represent the propagated standard deviation, calculated using the standard formula for error propagation.

## Results and discussion

### Design of triplex-controlled CRISPR–Cas12a system

**Figure 1.**
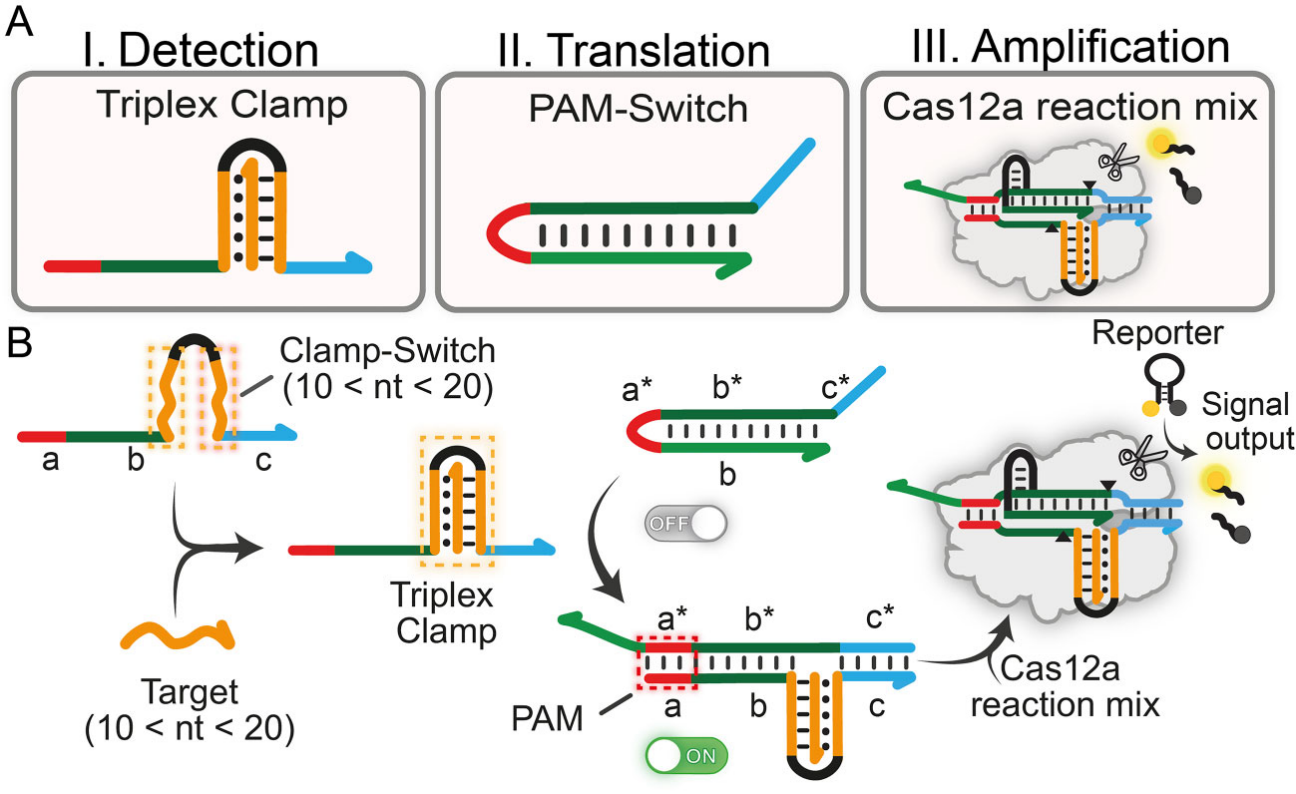
Design of triplex-based Cas12a detection system. (**A**) Schematic representation of the three functional modules required to generate the signal output. (**B**) Clamp-Switch adopts its folded triplex conformation only in the presence of a homopurine ssDNA/RNA target in solution. Target binding induces the formation of the Triplex Clamp, which subsequently triggers the hairpin strand displacement reaction (SDR). This reaction induces hairpin-to-duplex conformational change of the PAM-Switch module and PAM complementation, triggering Cas12a *trans*-cleavage activity and consequent generation of fluorescence output.

Our design involves two DNA modules engineered to trigger Cas12a activity only in the presence of a specific NA input: (i) a Clamp-Switch DNA probe for the recognition of the NA target of interest which folds into a triplex DNA only upon target binding (Triplex Clamp, Fig. 1A), and (ii) a specifically designed PAM-engineered DNA switch (i.e. PAM-Switch) that activates CRISPR–Cas12a in response to hairpin to duplex reconfiguration and PAM complementation. To monitor downstream collateral cleavage activity, a Cas12a reaction mix including the RNP complex and a FRET-based DNA reporter is added in solution (Fig. 1B). Specifically, we used a single-stranded hairpin DNA reporter bearing a fluorophore (FAM) and a quencher (BHQ1) at the two ends of the self-complementary portions to monitor *trans*-cleavage Cas12a activity [2]. The Clamp-Switch probe is composed of different functional motifs. Its core consists of two polypyrimidine portions (orange motifs, Fig. 1B): the first one recognizes the polypurine ssDNA/RNA target through WCF interactions (dashed lines), while the second one binds to duplex DNA via parallel Hoogsteen interactions (dotted lines) through a clamp-like switching mechanism. Thus, the formation of the Triplex Clamp is driven by a structure-switching mechanism, resulting in Clamp-Switch closure exclusively in the presence of the NA target with superior sensitivity than standard linear DNA probes (Supplementary Fig. S1). These two polypyrimidine motifs are separated by a flexible, unstructured 4-base loop and are flanked by two tail regions (indicated as a, b, and c) that are complementary to the corresponding motifs (a*, b*, and c*) of the PAM-Switch.

**Figure 2.**
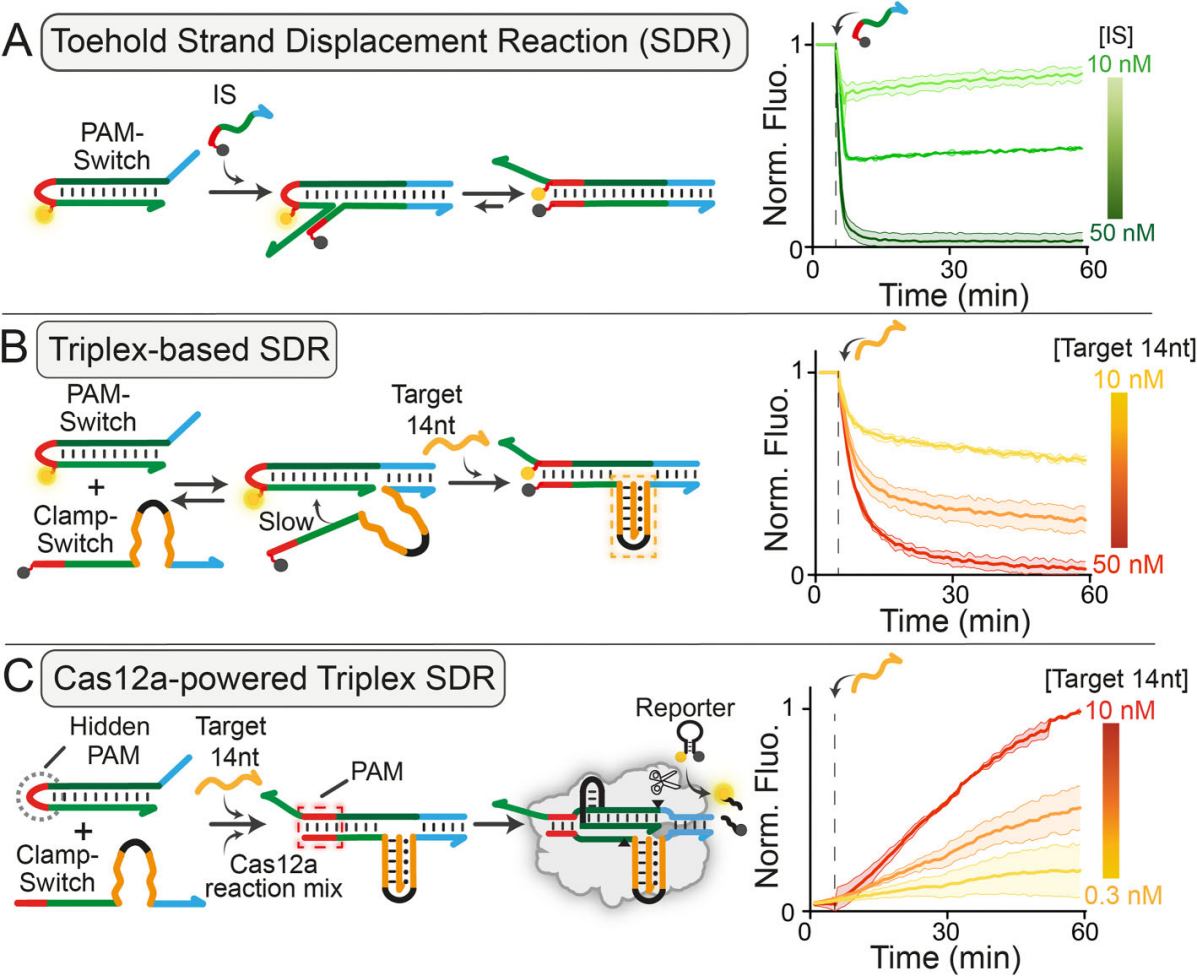
(**A**) Schematic representation of the toehold-based SDR (left). The FAM-labelled PAM-Switch probe (PAM-Switch_F, 50 nM) reconfigures into a duplex upon hybridization with a complementary invading BHQ1-labeled ssDNA target (Linear IS at 10, 30, or 50 nM). On the right, kinetic profiles of the toehold-based SDR. (**B**) Schematic representation of the triplex-based SDR where target-dependent Triplex Clamp formation leads to hairpin-to-duplex reconfiguration (left). On the right, kinetic profiles showing fluorescence quenching upon addition of Target 14 nt to a solution containing PAM-Switch (PAM-Switch_F, 50 nM) and Clamp-Switch (CS_Q 50 nM) at 37°C, confirming triplex-controlled strand displacement reacton. (**C**) Schematic representation of the triplex-based SDR controlling Cas12a activity (left). Triplex Clamp formation triggers strand displacement and enables PAM complementation (highlighted in red), thereby activating Cas12a binding and *trans*-cleavage. Kinetic profiles showing ssDNA detection when the reaction is combined with downstream Cas12a-based signal amplification (right). The Cas12a-based triplex SDR was conducted by adding different concentrations of Target 14 nt (0.3–1–10 nM) to a solution containing PAM-Switch (0.5 nM), Clamp-Switch (CS_14, 20 nM), and a Cas12a reaction mixture (20 nM RNP complex and 100 nM FRET-based DNA reporter). All experiments were carried out at 37°C in 10 mM Tris–HCl buffer solution containing 10 mM MgCl₂ and 50 mM NaCl at pH 7.0. For all experiments, error bars represent standard deviations from three independent replicates (for sequence details, see Supplementary Tables S1–3).

The second module consists of a DNA hairpin (PAM-Switch), in which the anti-PAM sequence (PAM*) is sequestered within a short loop connecting two self-complementary DNA domains targeted by Cas12a. This configuration includes a 20-base pair stem formed by the hybridization of two complementary strands: the target strand (TS) and the non-target strand (NTS), designated as domains a and a*, respectively (Fig. 1B). In its folded hairpin conformation, the PAM site remains inaccessible, thereby maintaining the Cas12a activity in an inactive state. We have recently demonstrated that by employing an optimized PAM-Switch design, it is possible to control Cas12a activation in response to external molecular cues as a means of hairpin-to-duplex reconfiguration [53].

Here, we take advantage of the proximity effect of the Triplex Clamp’s flanking portions (orange motifs, Fig. 1B) induced by target binding to trigger a toehold-mediated SDR on the PAM-Switch. The SDR is associated with a hairpin-to-duplex conformational change, which exposes the PAM motif, enabling Cas12a binding and activity. Specifically, a toehold motif of Clamp-Switch (c, light blue) complementary with the toehold region of the PAM-Switch probe is necessary to drive the SDR (c*, light blue). Upon triplex formation, a displacement domain (b, dark green, and a, red) of the Clamp-Switch complements the 20-base Cas12a targeting region of the PAM-Switch (b*, dark green) and the locked PAM* sequence (a*, red) (Fig. 1B). As a result, folded Triplex Clamp probe induces the reconfiguration of the PAM-Switch and activates Cas12a, generating a concentration-dependent *trans*-cleavage activity (Fig. 1B).

Notably, the NA target (orange strand, Fig. 1B) does not interact directly with the crRNA guide of the RNP complex but is instead recognized by the clamp region of the Clamp-Switch probe (c, orange portion in Fig. 1B), offering several advantages. First, it allows Cas12a activation in response to any triplex-forming homopurine NA sequence, eliminating the need for ssDNA sequence complementarity with the crRNA. Thus, our modular design may help achieve the detection of multiple targets using a single CRISPR reaction mixture [55]. By modifying the clamp region of the triplex probe—without the need to redesign the crRNA guide for each target—the system eliminates the need for different crRNAs and sequence-dependent *trans*-cleavage, thereby ensuring optimal *trans*-cleavage for any target [56]. Finally, the triplex structure provides a dual mechanism for target recognition, which results in increased specificity to single-base mutations compared to duplex DNA [30].

### Hairpin SDR controlled by Triplex Clamp formation

Toehold and displacement domains can function independently or in combination [57, 58], and the introduction of an unstructured loop between them can hinder the SDR on the time scale of the analysis [59, 60]. Our design leverages the key concept that target-induced triplex formation brings the toehold and displacement domains into close proximity, inducing strand invasion and hairpin-to-duplex reconfiguration of PAM-Switch. Thus, we studied Triplex Clamp formation using a Clamp-Switch (CS_FQ) with a 20 nt clamp region on each side modified with a fluorophore–quencher pair (FAM–BHQ1) at the termini of the clamp region. Binding assays with ssDNA targets of different lengths (from 10 to 20 nt) confirmed the triplex formation (Supplementary Fig. S2). Of note, as Hoogsteen interactions (both CG-C^+^ triplets and TA-T triplets) are sensitive to pH changes, also the observed binding affinity is pH-dependent (*K*_D, pH 6.5_ = 2 ± 1 nM, *K*_D, pH 7.0_ = 5 ± 1 nM, *K*_D, pH 7.5_ = 51 ± 7 nM, *K*_D, pH 8.0_ = 265 ± 1 nM, see Supplementary Fig. S3).

Then, we monitored toehold-mediated SDR using a FAM-tagged PAM-Switch (PAM-Switch_F). Specifically, we first compared the kinetic profiles of the reaction in the presence of a fully complementary DNA invading strand (IS, Fig. 2A) and a Clamp-Switch probe (CS_Q, Fig. 2B), both labeled with a 5′-end BHQ1. By adding the Target 14 nt in solution, we confirmed that the hairpin-to-duplex transition of the PAM-Switch probe depends only on triplex formation as fluorescence quenching is observed only in response to hairpin-to-duplex switch. Additionally, the kinetic profiles at tested concentrations are consistent with those relative to a linear IS, even though showing a slightly slower signal change over time due to Triplex Clamp folding needed to trigger the reaction. Next, we monitored the toehold-mediated SDR using a Cas12a reaction mix (RNP 20 nM, FRET-based DNA reporter 100 nM) as signal transducer, testing the system in the presence and absence of the Target 14 nt (Fig. 2C). As expected, we observed a concentration-dependent signal increase when the Target 14 nt was added in solution, as a consequence of triplex-driven SDR, PAM complementation and subsequent Cas12a activation. We performed different control experiments to confirm the proposed mechanism and the need for the DNA to trigger the reaction (Supplementary Fig. S4). For further optimization, we tested Clamp-Switch probes with different numbers of 5′- extra bases and varying toehold lengths (0, 6, 12, and 18 nt, Supplementary Fig. S5) [57]. Extra 5′-terminal nucleotides on the Clamp-Switch facilitate efficient hairpin-to-duplex transition as expected by the thermodynamics of hairpin SDRs. As expected, CRISPR-powered strand displacement controlled by triplex formation exhibits higher sensitivity (LOD = 0.3 nM, Fig. 2C), enabling detection of target concentrations one order of magnitude lower than those obtained using either toehold-based and triplex-based SDR (Fig. 2A and B). Of note, the pM sensitivity aligns with that reported for pre-amplification free Cas12a-based assays [61, 62]. It is important to note that the kinetics profiles of Cas12a-powered SDRs, however, show slower signal generation over time compared to that reported in Fig. 2A and B. This is likely due to the equilibration time required to achieve molecular reconfiguration of the involved probes (i.e. target binding, triplex folding and subsequent hairpin-to duplex switch) and subsequent Cas12a binding/activation.

### Optimization of triplex-based Cas12a assay for ssDNA detection

**Figure 3.**
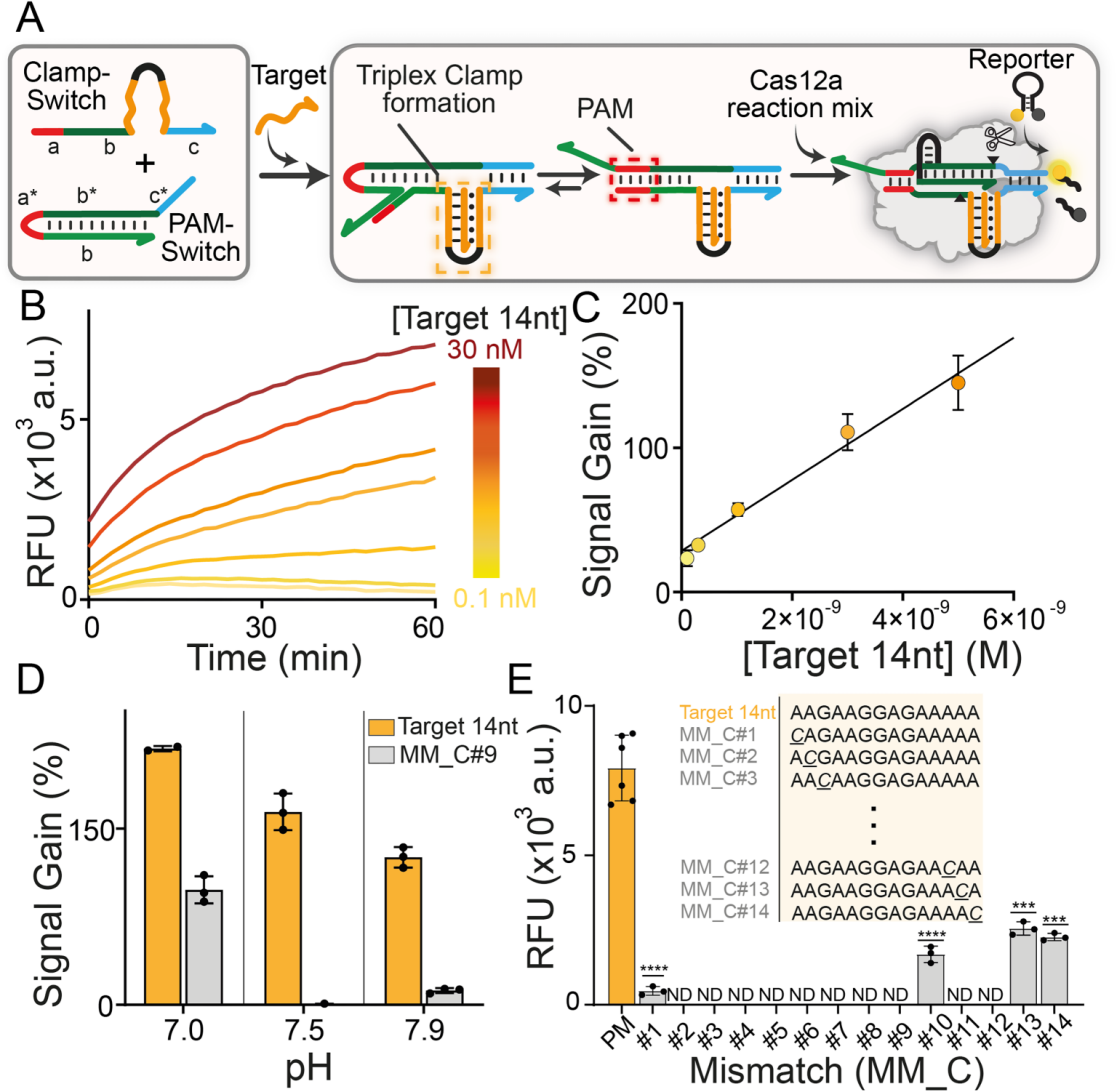
Proof-of-concept demonstration of highly specific Cas12a-powered detection of ssDNA using Triplex Clamp structure. (**A**) Schematic representation of the triplex-based Cas12a detection system. (**B**) Time-dependent fluorescence kinetic analysis showing the detection of increasing concentrations (0.1, 0.3, 1, 3, 5, 10, and 30 nM) of Target 14 nt. (**C**) Linear dynamic range (between 0.1 and 5 nM; *R*^2^ = 0.9522, *n* = 3) of Target 14 nt concentration obtained by plotting fluorescence signal change versus target concentration. Signal gain values (%) were calculated after 15 min of cleavage activity and represent the relative fluorescence signal change associated with collateral cleavage activity due to the addition of Target 14 nt. (**D**) Specificity study performed at three different pH values using 5 nM of a single-nucleotide mismatch (MM_C#9) and Target 14 nt perfect match. Signal gain values (%) were calculated after 15 min cleavage reaction. All the values reported show the mean ± SD, where *n* = 3 replicates. (**E**) Bar graphs showing the difference between the fold change with respect to the perfect match (Target 14 nt, 5 nM) and to the specific single-nucleotide mismatch (MM_C) target (5 nM). All the values reported show the mean ± SD, where *n* = 3 replicates. Asterisks indicate statistical significance relative to the PM (perfect match) sequence, calculated using an unpaired *t*-test with Welch’s correction (*n* = 3): *P* < 0.0001 (^****^), *P* < 0.001 (***), *P* < 0.01 (**), and *P* ≤ 0.05 (*). ND (non-detectable) indicates values below the blank signal, considered not detectable in this assay.

To optimize our detection system, we used a ssDNA Target of 14 nt as a model target (Target 14 nt, see Supplementary Table S3 in the Supplementary Information). Of note, the Cas12a detection system requires DNA targets of at least 17 nt in length for the direct activation of *trans*-cleavage activity [1]. Here, we aim to demonstrate that shorter ssDNA can also be used to control Cas12a detection systems (Fig. 3A). To demonstrate this, we first optimized the Cas12a-based assay conditions in terms of PAM-Switch (0.5 nM) and Clamp-Switch (20 nM) concentrations (Supplementary Figs S6 and S7). Under such experimental conditions, a rapid activation of the Cas12a detection system is observed over time in response to the addition of Target 14 nt (Fig. 3B) with picomolar sensitivity (LOD = 791 pM, Fig. 3C). Then, we performed specificity tests using variant sequences of Target 14 nt having a single base mutation at different positions. Of note, affinity-based binding assays using a FAM–BHQ1 Clamp-Switch (CS_FQ) confirmed that Triplex Clamp formation is highly specific for single-base mutations (Supplementary Fig. S8A). Since pH strongly affects Hoogsteen interactions and triple helix stability, the specificity can be easily tuned by changing the pH of the solution (Supplementary Fig. S8B). To investigate the overall specificity of the triplex-based Cas12a detection system, we tested three different pH values (pH 7.0, 7.5, and 7.9). Our data reflect the pH-dependent folding of Triplex Clamp, showing a different detection specificity when the Triplex Clamp folding is integrated into the CRISPR–Cas12a recognition system (Fig. 3D). Specifically, we observe an increased specificity at higher pH values (7.5 and 7.9), consistent with the lower stability of the Triplex Clamp structure under mildly alkaline conditions. This behavior reflects the intrinsic characteristics of triplex DNA, where even a single-base mismatch can substantially impair triplex formation—an effect that is more pronounced at higher pH values due to the reduced protonation of cytosines required for stable CG-C^+^ triplet interactions. The reduced protonation weakens hydrogen bonding, destabilizing the triplex structure. As a result, the combination of mismatch-induced destabilization and pH-dependent triplex dissociation synergistically enhances the system’s capacity to discriminate single-nucleotide variants (SNVs) at slightly alkaline pH values [63]. To demonstrate this, we performed specificity assays by screening ssDNA targets containing either single-base C (i.e. MM_C variants) or T (MM_T variants) mismatches at each position along the Target 14 nt sequence, confirming high specificity for the tested targets. As expected, a central mismatch in the triplex destabilizes the formation of the triple helix more than the presence of the mutation in the terminal positions of the sequence (Fig. 3E). In addition, a G→C substitution results in a further enhancement of specificity compared to an A→T substitution (Supplementary Fig. S9). To further probe the robustness of our platform under non-ideal conditions, we assessed the system’s ability to maintain high specificity in the presence of a high molar excess of nonspecific DNA target (Target 14 nt_2MM, Supplementary Fig. S10). Finally, we investigated the effect of 5′- or 3′ -overhangs of Target 14 nt (Supplementary Table S3 and Supplementary Fig. S11) on the detection platform. Extra nucleotides at the 5′ end led to a length-dependent decrease of fluorescence transduction (Supplementary Fig. S11A), likely due to steric hindrance effects on the toehold-mediated SDR between the Triplex Clamp and the PAM-Switch. However, the system can detect the DNA input also in the presence of 5′ -overhangs. Additional nucleotides at the 3′ end show no significant impact on the signal transduction, except in the case of extensions longer than 30 nt, where a modest decrease in signal intensity is observed (Supplementary Fig. S11B).

### Triplex-controlled Cas12a detection regulated by ssDNA and RNA targets of different lengths

**Figure 4.**
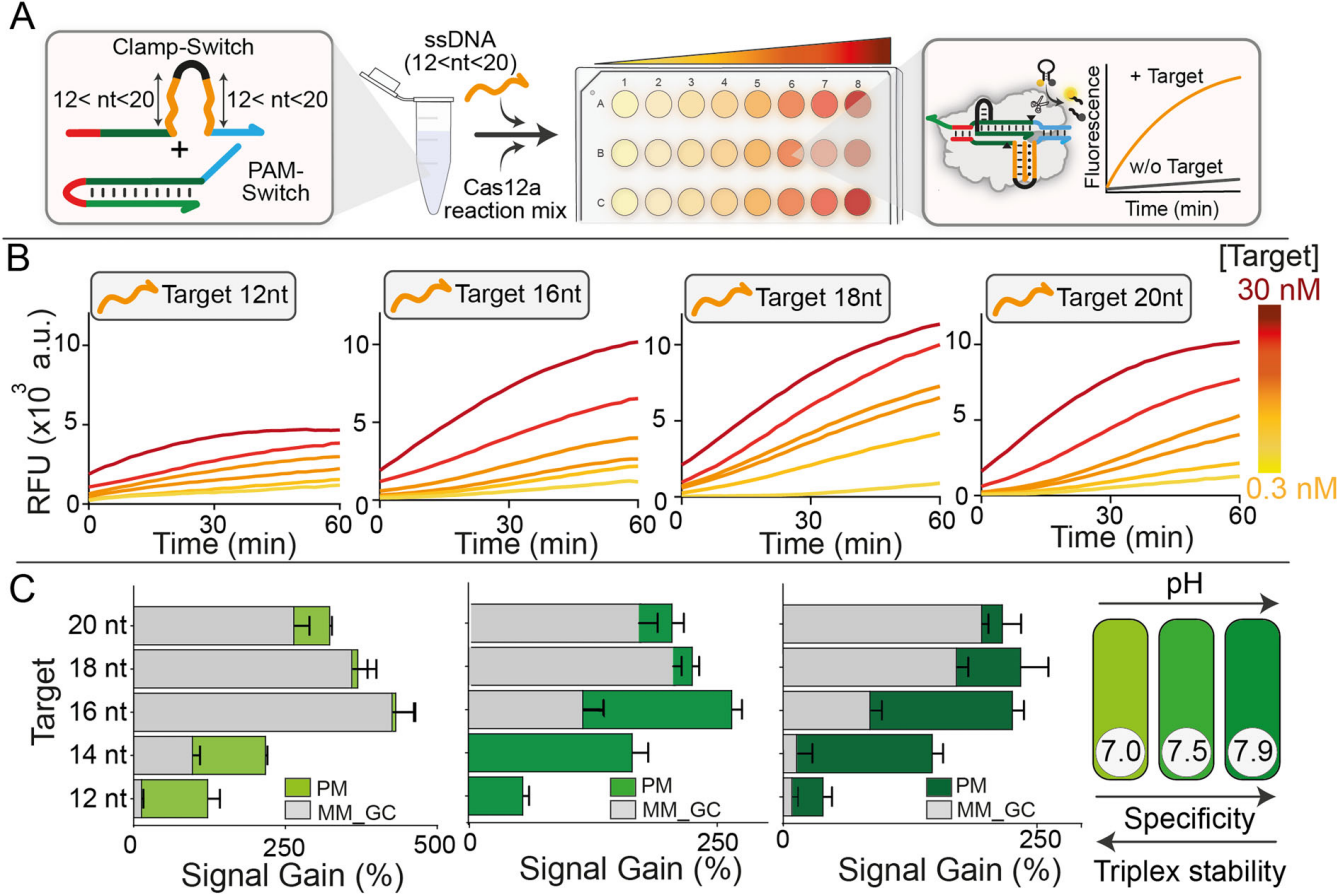
**A**) Schematic description of the proposed triplex-based CRISPR assay for the detection of ssDNA targets of different lengths (from 12 nt to 20 nt) by simply changing the Clamp-Switch probe using the same Cas12a reaction mix. (**B**) Time-dependent fluorescence kinetic analysis of Cas12a collateral cleavage at increasing concentrations of ssDNA targets. The experiments were conducted by adding different concentrations of ssDNA target (0.3, 1, 3, 5, 10, and 30 nM) to a solution containing PAM-Switch (0.5 nM) and the specific Clamp-Switch (20 nM, see Supplementary Table S2), and incubating at 37 °C for 15 min. These solutions were then mixed with the Cas12a reaction mix (RNP complex 20 nM, FRET-based DNA reporter 100 nM) previously incubated at 37°C for 30 min. All the experiments were conducted in a buffer containing 10 mM Tris–HCl, 50 mM NaCl, and 10 mM MgCl_2_, pH 7.0. (**C**) Superimposed bar graphs show fluorescence signal intensities generated by the perfect match (PM) and single-nucleotide mismatch (MM) sequences (from left to right, Target 12 nt_MM_GC#5, Target 14 nt_MM_C#7, Target 16 nt_MM_GC#8, Target 18 nt_MM_GC#10, and Target 20 nt_MM_GC#12 at 5 nM), measured at *t* = 15 min under three different pH conditions (pH 7.0, 7.4, and 7.9, from left to right). For each condition, 10 nM of ssDNA target (PM or MM) was introduced into a solution containing PAM-Switch (0.5 nM) and Clamp-Switch (20 nM). Separately, the Cas12a reaction mixture was preincubated at 37 °C for 30 min and subsequently added to the target-containing solutions. Reactions were carried out in a 10 mM Tris–HCl buffer solution containing 50 mM NaCl and 10 mM MgCl_2_. Fluorescence values represent the mean ± SD of three independent replicates (*n* = 3). Detailed sequence and mismatch position information for PM and MM targets are provided in Supplementary Table S3.

To generalize the approach, we tested the platform using ssDNA targets of varying lengths by simply changing the clamp portion of the Clamp-Switch DNA probe responsible for target recognition (Fig. 4A, see Supplementary Tables S2 and S3). Kinetic fluorescence analysis demonstrates that our triplex-based platform enables the detection of homopurine ssDNA targets of different lengths (from 12 to 20 nt) (Fig. 4B). We also performed specificity assays using mutated variants (i.e. C-type point mutations in the middle of the sequence) by collecting the signal of the perfect match target (PM, 10 nM) and the corresponding signal of mutated target (MM, 10 nM) at pH values comprised between pH 7.0 and 7.9. Our results confirm enhanced detection specificity under slightly alkaline pH values for all the ssDNA target lengths tested (Fig. 4C). Notably, the specificity improves as the length decreases, as a single base mutation has a higher impact on the overall free energy of triplex folding. Additionally, as proof of concept for the potential applicability of our approach for diagnostic applications, we assessed the system’s ability to detect a synthetic genomic sequence. Specifically, we selected a 14-base target sequence derived from the *Streptococcus pneumoniae* TIGR4 genome (sequence details in Supplementary Tables S2 and S3, and Supplementary Fig. S12), demonstrating that our method is not limited to specific targets but can, in principle, be used to detect natural viral or bacterial genomes presenting homopurine motifs. To further generalize the proposed method, we also demonstrated the possibility of combining our triplex-based hybridization network with an isothermal pre-amplification step to achieve ultrasensitive detection using nicking enzyme-assisted amplification (LOD = 10 fM, Supplementary Fig. S13). Of note, this isothermal DNA amplification generates ssDNA amplicon and thus can be easily combined with our triplex-based detection systems.

Comparison of the triplex-controlled Cas12a detection system with previously reported Cas12a-based assays (Table 1) indicates that our pre-amplification-free approach shows similar sensitivity but superior specificity, owing to its ability to discriminate SNVs within ssDNA targets—a feature typically limited in Cas12a detection systems. Importantly, by decoupling target recognition from guide RNA hybridization, our design confers superior versatility, enabling the same CRISPR–Cas12a complex to detect both ssDNA and RNA targets of varying lengths.

**Table 1.** Comparison between Cas12a-based detection platforms and our triplex-based system

CRISPR enzyme	Input	Compatible with amplification	Time	LODs	Specificity (SNV on dsDNA)	Specificity (SNV on ssDNA/RNA)	CRISPR–RNA	Ref.
*Lb*Cas12a (DETECTR)	dsDNA,ssDNA	Yes (RPA)	∼2 h	116 copies/ul	Limited to seed region	Aspecific	Sequence specific	[1]
*Lb*Cas12a (HOLMES)	dsDNA,RNA	Yes(PCR, RT-PCR)	∼1 h	10 aM	Limited to seed region	–	Sequence specific	[3]
*Lb*Cas12a	RNA	Yes(RT-RPA)	∼30 min	<fM	–	ND	Sequence specific	[10]
*Lb*Cas12a	RNA	No	60 min	132–767 pM	–	High	Sequence specific	[13]
*Lb*Cas12a	ssDNA	No	90 min	29 pM	–	Low	Sequence specific	[64]
*Lb*Cas12a	dsDNA	Yes(NEAA)	∼20 min	80 CFU/ml	ND	–	Sequence specific	[65]
*Lb*Cas12a	ssDNA,RNA	Yes(NEAA)	∼30 min	fM	ND	High	Sequence independent	Our work

**Figure 5.**
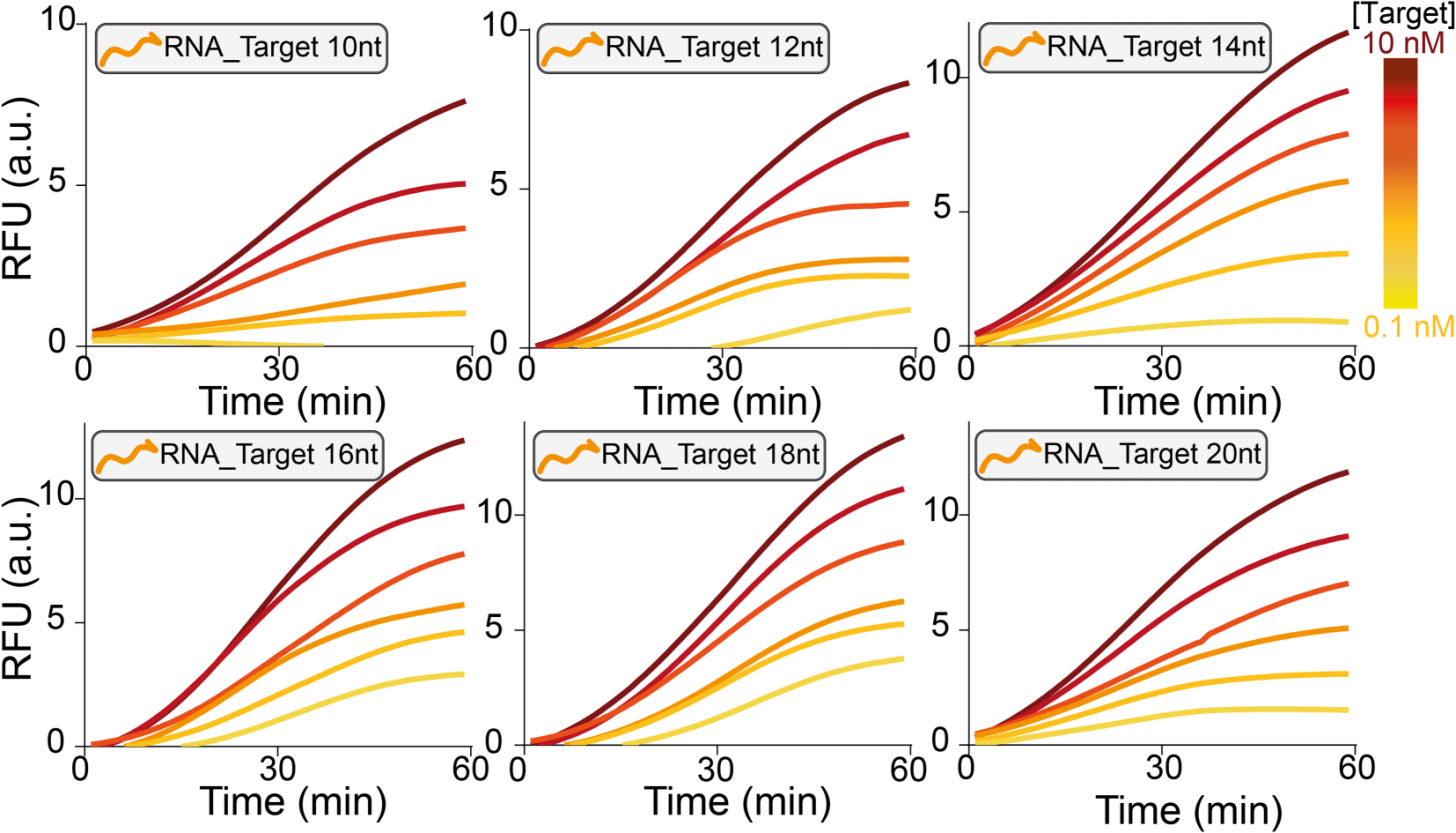
Kinetic profiles of the RNA triplex-based activation of the Cas12a detection system. Experiments were performed using different concentrations (0.1, 0.3, 1, 3, 5, and 10 nM) of RNA targets of different lengths (from 10 to 20 nt). RNA targets were introduced into a buffer solution (10 mM Tris–HCl 50 mM NaCl 10 mM MgCl_2_ at pH 7.0) containing PAM-Switch (0.5 nM) and CS_ RNA (20 nM). Following a 15-min incubation at 37 °C, a Cas12a reaction mixture (FRET-based DNA reporter 100 nM + RNP complex 20 nM) was added, and fluorescence signal acquisition was started immediately.

Finally, we have adapted the design for the detection of RNA targets. It is well-established that Cas12a can naturally recognize and discriminate dsDNA targets, making its adaptation for RNA detection challenging. To achieve this goal, we modified the clamp region of the Clamp-Switch probe (see Fig. 1, orange portions), generating a hybrid RNA–DNA Clamp-Switch probe (CS_RNA) that features the two clamp regions made of RNA. The hybrid RNA–DNA probe is necessary to enable the formation of the specific Clamp Triplex, as DNA-based clamp probes cannot fold into a triplex structure in the presence of RNA targets as this configuration is thermodynamically unstable [39]. By doing so, we are able to detect RNA targets, of various lengths (10–20 nt) with a sensitivity in the picomolar range (Fig. 5).

### “Well-resolved” ssDNA target detection using a single Cas12a-based reaction mix in an array-based fluorescence platform

**Figure 6.**
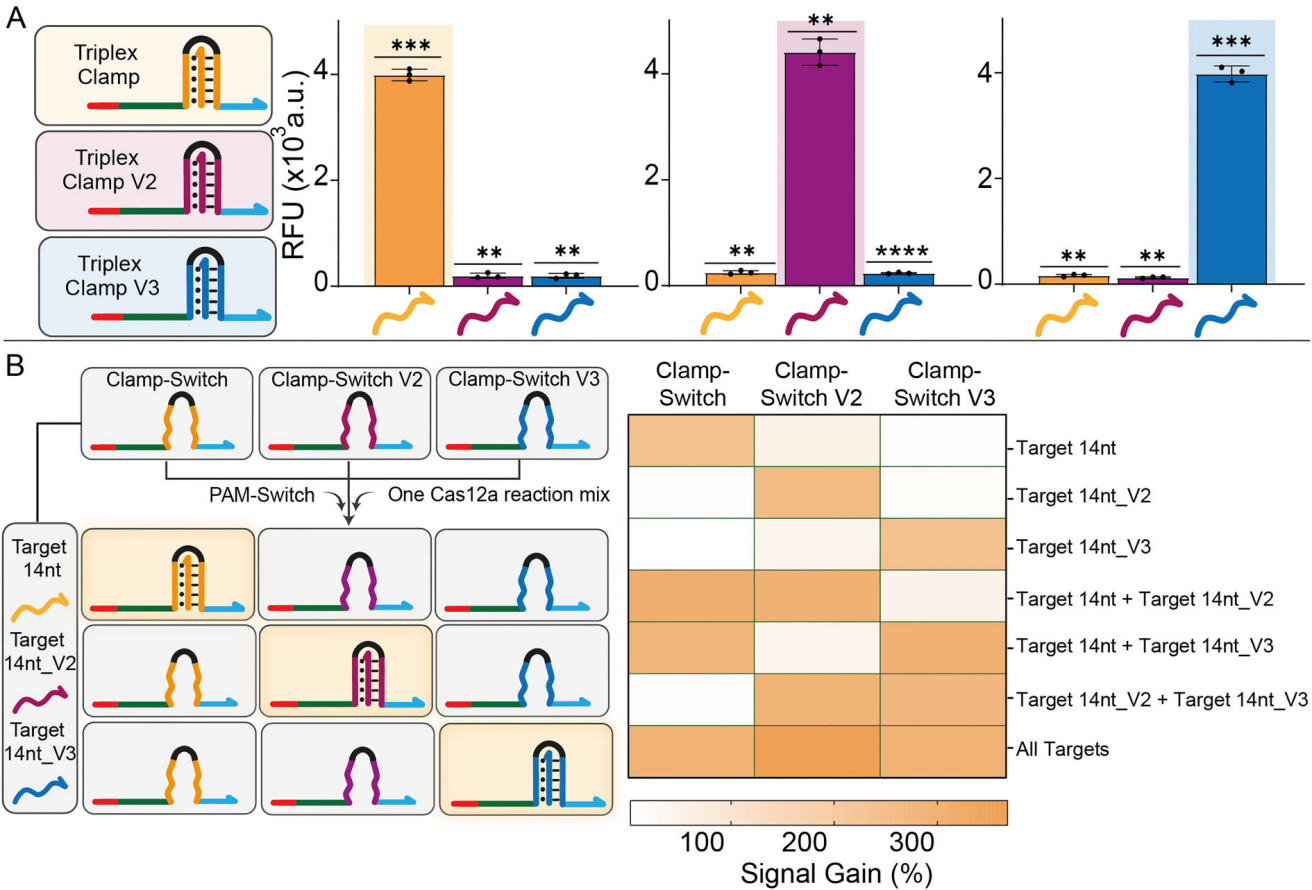
Multiple detection of ssDNA targets using a well-array platform. (**A**) Bar graph depicting the sequence-dependent and highly specific interactions between various ssDNA targets and their corresponding clamp domains, leading to the activation of Cas12a collateral cleavage activity. Probe complementarity is highlighted using matched color coding. Experiments were conducted at 37 °C by adding a CRISPR–Cas12a reaction mix (20 nM RNP complex and 100 nM FRET-based DNA reporter) to a buffer solution (10 mM Tris–HCl 50 mM NaCl and 10 mM MgCl_2_ at pH 7.0) containing PAM-Switch (0.5 nM), the specific Clamp-Switch (20 nM) and ssDNA targets (Target 14 nt, Target 14 nt_V2, Target 14 nt_V3; 5 nM), pre-incubated at 37 °C for 15 min. The reported RFU were measured after 15 min of reaction and represent the change in fluorescence signal induced by Cas12a *trans*-cleavage in the presence and absence of the ssDNA target. Data are expressed as mean ± standard deviation (SD), *n* = 3 replicates. Statistical analysis was performed using a two-tailed unpaired *t*-test with Welch’s correction. Asterisks indicate statistical significance relative to the background signal: *P* < 0.0001 (^****^), *P* < 0.001 (***), *P* < 0.01 (**), and *P* ≤ 0.05 (*). (**B**) Schematic overview of the experimental workflow (left) and bar graph (right) showing the Signal Gain (%) in response to different combinations of the three ssDNA targets (see Supplementary Information for details). Signal Gain (%) was calculated after 15 min of cleavage reaction. Values are reported as mean ± SD, *n* = 3 replicates.

Since our strategy does not require modification of the RNP complex to recognize different NA targets, we further investigated the potential for parallel NA analysis using a single Cas12a reaction mix. As a proof-of-concept demonstration, we designed two additional Clamp-Switch probes (designated V2 and V3), each containing a unique clamp motif (purple for V2 and blue for V3; Fig. 6A) that selectively binds to its corresponding ssDNA target. Notably, the target sequences for V2 and V3 (Target 14 nt_V2 and Target 14 nt_V3; see Supplementary Table S3) consist of scrambled homopurine sequences that ensure target-specific activation of Cas12a. Each of the three ssDNA targets (Target 14 nt, Target 14 nt_V2, and Target 14 nt_V3) was successfully detected using the same RNP complex thanks to the specificity of the corresponding Clamp-Switch modules (Fig. 6A, right; Supplementary Fig. S14). To assess the capacity to discriminate between different DNA targets, we implemented a parallel detection format using standard 96-well plates (25 µl per well), where each well contains a distinct Clamp-Switch probe. This setup allows the simultaneous assessment of multiple Cas12a-based signal transduction controlled by target–probe combinations under identical experimental conditions. The resulting heatmap demonstrates the system’s capacity to detect and distinguish each target with high specificity and without significant cross-reactivity (Fig. 6B). Indeed, when only one ssDNA target is present in the reaction mix, fluorescence can be observed exclusively in the well containing the matching Clamp-Switch probe. On the contrary, when multiple targets are mixed in the same well, knowing the specific Clamp-Switch probe that is present in solution allows unambiguous identification of each individual ssDNA target. This setup enables parallel screening of multiple NA targets with a single, unmodified Cas12a reaction mix without the need for target-specific RNPs. Importantly, signal intensities and response times remained consistent and robust even in the presence of noncognate targets, highlighting the scalability and efficiency of this detection approach for NA sensing.

## Conclusion

In this study, we report a strategy to control CRISPR–Cas12a activation through a NA-based hybridization network triggered by the formation of a clamp-like triplex DNA. We show that Cas12a activity can be modulated via a triplex-based strand displacement system that drives the conformational change of a PAM-Switch probe from a hairpin to a duplex state. This transition enables PAM complementation and consequent Cas12a activation. Our approach allows Cas12a activation without requiring complementarity between the crRNA sequence and the target NA. This results in the use of a single RNP complex for the detection of multiple distinct oligonucleotide sequences. Specifically, we show that it is possible to detect multiple ssDNA and RNA targets, achieving picomolar sensitivity using the same Cas12a reaction mix. The sensing platform is flexible as it expands detection to oligonucleotides shorter than those typically required for direct Cas12a-based activation (17 nt). Furthermore, by using a clamp-like mechanism to bind the target, it also overcomes one of the major limitations of standard Cas12a diagnostics—namely, the difficulty in discriminating SNVs. Our system indeed enables precise discrimination between single-base mismatched (MM) and perfectly matched (PM) sequences, thanks to the enhanced selectivity of the Triplex Clamp probe.

Despite these promising results, our approach presents specific limitations to be addressed. First, as expected, by increasing the target length (i.e. from 16 to 20 nt), the specificity of the triplex folding is slightly lower, making the overall specificity of the Cas12a-based detection system optimal when using shorter target lengths (comprised between 12 and 16 nt). Second, the approach relies on the presence of a homopurine sequence in the NA target to support triple helix formation. While such homopurine-rich regions are found in various bacterial and viral genomes [66, 67], this structural requirement inevitably restricts the range of sequences that can be targeted and may limit the assay’s universality. However, the platform’s inherent modularity, along with its capacity to detect different target lengths—including those below the length threshold typically required for Cas12a activation—may enable the simultaneous detection of multiple distinct homopurine-rich motifs within the same gene, even in the presence of closely related sequences, thus expanding the diagnostic potential. By integrating structure switching via programmable triplex formation, the system achieves enhanced sequence specificity, increased tolerance to short targets, and modular multiplexing capabilities, thus addressing several of the intrinsic challenges relative to Cas12a-based sensing. Taken together, these features may broaden the scope of CRISPR–Cas12a applications in NA diagnostics—particularly in scenarios requiring single-nucleotide resolution, such as clinical diagnostics, pathogen detection, and precision medicine. Moreover, the conditional control strategy demonstrated here could be generalized to other CRISPR systems, paving the way for more adaptable and intelligent molecular tools in nanomedicine and synthetic biology.

## Supplementary Material

gkaf1392_Supplemental_File

## Data Availability

All data are available in the manuscript or in the Supplementary Information. Materials that support the findings of this study are available from the corresponding author upon request.

## Notes

Present address: Nanobioelectronics & Biosensors Group, Catalan Institute of Nanoscience and Nanotechnology(ICN2), CSIC and BIST, Campus UAB, Bellaterra 08193 Barcelona, Spain
