# Supplementary material for: Triplex DNA clamp regulates Cas12a activation for ssDNA and RNA sensing
Source: Nucleic Acids Res. 2026 Jan 8;54(1):gkaf1392. doi: 10.1093/nar/gkaf1392 (PMC12781877; doi:10.1093/nar/gkaf1392)
Supplement: gkaf1392_Supplemental_File [file gkaf1392_supplemental_file.docx]

Supplementary Information

**Triplex DNA Clamp Regulates Cas12a Activation for ssDNA and RNA Sensing**

Andrea Celeste Di Pede^1^, Neda Bagheri^1^, Erica Belforte^1^, Alessio Palone^1^, Marianna Rossetti^1^ and Alessandro Porchetta^1,*^

1 Department of Chemical Science and Technologies, University of Rome, Tor Vergata, Via della Ricerca Scientifica 1, 00133 Rome, Italy

* To whom correspondence should be addressed. Tel. +39 06 7259 4461;

Email. [alessandro.porchetta@uniroma2.it](mailto:alessandro.porchetta@uniroma2.it).

**Table *S*1. List of the PAM-Switch probes**

| Name | Sequence (5’→3’)^a^ |  |
| --- | --- | --- |
| PAM-Switch_Toehold0 | *AGA TTT AGC CAT GTG TAG ACC AAA GT*C TAC ACA TGA CTA AAT CT | |
| PAM-Switch_Toehold6 | **CCT ATC** *AGA TTT AGC CAT GTG TAG ACC AAA GT*C TAC ACA TGA CTA AAT CT | |
| PAM-Switch | **TCT TAC CCT ATC** *AGA TTT AGC CAT GTG TAG ACC AAA GT*C TAC ACA TGA CTA AAT CT | |
| PAM-Switch_Toehold18 | **CTC TTCT CTT ACC CTA TC***A GAT TTA GCC ATG TGT AGA CCA AAG T*CT ACA CAT GAC TAA ATC T | |
| PAM-Switch_F | **TCT TAC CCT ATC** *AGA TTT AGC CAT GTG TAG ACC AAA*(6-FAM)*GT*C TAC ACA TGA CTA AAT CT | |

a *Italic* bases represent the Cas12-targeting region. **Bold** bases indicate the toehold region of the PAM-Switch probe. *Italic* and bold bases are complementary either to the linear invading strand (Linear IS) and to the folded Triplex Clamp.

**Table S2. List of the Clamp-Switch probes (CS)**

| Name | Sequence (5’→3’)^a^ |  |
| --- | --- | --- |
| CS_FQ | (6-Fam)-T TCT TCT TTC TTC CTC TTT TTT TTT TTT TTC TCC TTC TTT CTT CTT- (BHQ-1) | |
| CS_Q | (BHQ-1)-T *TAG ACT TTG GTC TAC ACA TGG CTA AAT CT*A TTC TTC CTC TTT TTT TTT TTT TTC TCC TTC TTA *GAT AGG GTA AGA* | |
| CS_Target 12nt | *AGA CTT TGG TCT ACA CAT GGC TAA ATC TA*C TTC CTC TTT TTT TTT TTT TTC TCC TTC A*GA TAG GGT AAG A* | |
| CS_Target 14nt | *AGA CTT TGG TCT ACA CAT GGC TAA ATC TA*T TCT TCC TCT TTT TTT TTTT TTT CTC CTT CTT A*GA TAG GGT AAG A* | |
| CS_*Sp* TIGR4 | *AGA CTT TGG TCT ACA CAT GGC TAA ATC TA*T TTC TTT TCC TCT CTT TTC TCT CCT TTT CTT T*AG ATA GGG TAA GA* | |
| CS_Target 16nt | *AGA CTT TGG TCT ACA CAT GGC TAA ATC T*AC TTT CTT CCT CTT TTT TTT TTT TTT CTC CTT CTT TCA *GAT AGG GTA AGA* | |
| CS_Target 18nt | *AGA CTT TGG TCT ACA CAT GGC TAA ATC T*AT TCT TTC TTC CTC TTT TTT TTT TTT TTC TCC TTC TTT CTT A*GA TAG GGT AAG A* | |
| CS_Target 20nt | *AGA CTT TGG TCT ACA CAT GGC TAA ATC T*AT CTT CTT TCT TCC TCT TTT TTT TTT TTT TCT CCT TCT TTC TTC TA*G ATA GGG TAA GA* | |
| CS_Target 14nt_V2 | *AGA CTT TGG TCT ACA CAT GGC TAA ATC T*AC CTT TTT CTT CCT TTTT TTT CCT TCT TTT TCC A*GA TAG GGT AAG A* | |
| CS_Target 14nt_V3 | *AGA CTT TGG TCT ACA CAT GGC TAA ATC T*AC TTT CCT TTT TTC TTT TTT CTT TTT TCC TTT CA*G ATA GGG TAA GA* | |
| CS_Extra#0 | *TTT GGT CTA CAC ATG GCT AAA TCT* ATT CTT CCT CTT TTT TTT TTT TTT CTC CTT CTT A*GA TAG GGT AAG A* | |
| CS_Extra#4 | *GTG TAG ACT TTG GTC TAC ACA TGG CTA AAT CT*A TTC TTC CTC TTT TTT TTT TTT TTC TCC TTC TTA *GAT AGG GTA AGA* | |
| CS_Extra#8 | *TCA TGT GTA GAC TTT GGT CTA CAC ATG GCT AAA TCT* ATT CTT CCT CTT TTT TTT TTT TTT CTC CTT CTT A*GA TAG GGT AAG A* | |
| CS_Toehold_0 | *AGA CTT TGG TCT ACA CAT GGC TAA ATC T*AT TCT TCC TCT TTT TTT TTT TTT TCT CCT TCT TA | |
| CS_Toehold_6 | *AGA CTT TGG TCT ACA CAT GGC TAA ATC T*AT TCT TCC TCT TTT TTT TTT TTT TCT CCT TCT TA*G ATA GG* | |
| CS_RNA | *AGA CTT TGG TCT ACA CAT GGC TAA ATC T*AU CUU CUU UCU UCC UCU UUU UUU UUU UUU UCU CCU UCU UUC UUC UA*G ATA GGG TAA GA* | |

a **Bold** bases represent the portion of the Clamp-Switch module that recognize the DNA or RNA target (orange line in Figure 1). The underlined bases refer to the loop portion of the Clamp-Switch (black portion in Figure 1) and the *italic* bases represent the portion of the probe that is complementary to the PAM-Switch.

**Table S3. List of ssDNA and RNA targets**

| **Name** | **Sequence (5’→3’)^a^** |
| --- | --- |
| Target 10nt | AGG AGA AAA A |
| Target 12nt | GAA GGA GAA AAA |
| Target 14nt | AAG AAG GAG AAA AA |
| Target 14nt_V2 | GGA AAA AGA AGG AA |
| Target 14nt_V3 | GAA AGG AAA AAA GA |
| Target 14nt_Extra#10 (5’)  Target 14nt_Extra#20 (5’)  Target 14nt_Extra#30 (5’) | TTA AAA TTT GAA GAA GGA GAA AAA  TTA AGT AAT GTT AAA ATT TGA AGA AGG AGA AAA A  TGT CCG GGG ATT AAG TAA TGT TAA AAT TTG AAG AAG GAG AAA AA |
| Target 14nt_Extra#10 (3’) | AAG AAG GAG AAA AAT TAA TCT TTG |
| Target 14nt_Extra#20 (3’) | AAG AAG GAG AAA AAT TAA GTC CCA CCA AAC AAT G |
| Target 14nt_Extra#30 (3’) | AAG AAG GAG AAA AAT GTC CGG GGA TTA AGT AAT GTT AAA ATT TG |
| *Sp* TIGR4 | AAA GAA AAG GAG AGT AGT ATG TAT |
| Target 16nt | GAA AGA AGG AGA AAA A |
| Target 18nt | AAG AAA GAA GGA GAA AAA |
| Target 20nt | AGA AGA AAG AAG GAG AAA AA |
| RNA_Target 10nt | AGG AGA AAA A |
| RNA_Target 12nt | GAA GGA GAA AAA |
| RNA_Target 14nt | AAG AAG GAG AAA AA |
| RNA_Target 16nt | GAA AGA AGG AGA AAA A |
| RNA_Target 18nt | AAG AAA GAA GGA GAA AAA |
| RNA_Target 20nt | AGA AGA AAG AAG GAG AAA AA |
| Target 12nt_MM_GC#5 | GAA G***C***A GAA AAA |
| Target 16nt_MM_GC#8 | GAA AGA A***C***G AGA AAA A |
| Target 18nt_MM_GC#10 | AAG AAA GAA ***C***GA GAA AAA |
| Target 20nt_MM_GC#12 | AGA AGA AAG AA***C*** GAG AAA AA |
| Target 14nt_MM_C#1 | ***C***AG AAG GAG AAA AA |
| Target 14nt _MM_C#2 | A***C***G AAG GAG AAA AA |
| Target 14nt _MM_C#3 | AA***C*** AAG GAG AAA AA |
| Target 14nt _MM_C#4 | AAG ***C***AG GAG AAA AA |
| Target 14nt _MM_C#5 | AAG A***C***G GAG AAA AA |
| Target 14nt _MM_C#6 | AAG AA***C*** GAG AAA AA |
| Target 14nt _MM_C#7 | AAG AAG ***C***AG AAA AA |
| Target 14nt _MM_C#8 | AAG AAG G***C***G AAA AA |
| Target 14nt _MM_C#9 | AAG AAG GA***C*** AAA AA |
| Target 14nt _MM_C#10 | AAG AAG GAG ***C***AA AA |
| Target 14nt _MM_C#11 | AAG AAG GAG A***C***A AA |
| Target 14nt _MM_C#12 | AAG AAG GAG AA***C*** AA |
| Target 14nt _MM_C#13 | AAG AAG GAG AAA ***C***A |
| Target 14nt _MM_C#14 | AAG AAG GAG AAA A***C*** |
| Target 14nt _MM_T#1 | ***T***AG AAG GAG AAA AA |
| Target 14nt _MM_T#2 | A***T***G AAG GAG AAA AA |
| Target 14nt _MM_T#3 | AA***T*** AAG GAG AAA AA |
| Target 14nt _MM_T#4 | AAG ***T***AG GAG AAA AA |
| Target 14nt _MM_T#5 | AAG A***T***G GAG AAA AA |
| Target 14nt _MM_T#6 | AAG AA***T*** GAG AAA AA |
| Target 14nt _MM_T#7 | AAG AAG ***T***AG AAA AA |
| Target 14nt _MM_T#8 | AAG AAG G***T***G AAA AA |
| Target 14nt _MM_T#9 | AAG AAG GA***T*** AAA AA |
| Target 14nt _MM_T#10 | AAG AAG GAG ***T***AA AA |
| Target 14nt _MM_T#11 | AAG AAG GAG A***T***A AA |
| Target 14nt _MM_T#12 | AAG AAG GAG AA***T*** AA |
| Target 14nt _MM_T#13 | AAG AAG GAG AAA ***T***A |
| Target 14nt _MM_T#14 | AAG AAG GAG AAA A***T*** |
| Target 14nt_2MM | AAG AAG G***TC*** AAA AA |
| Linear IS | (BHQ1)-TTA GAC TTT GGT CTA CAC ATG GCT AAA TCT GAT AGG GTA AGA |
| Duplex control probe | (6-FAM)-TTT TTC TCC TTC TT-(BHQ1) |
|  |  |

a Italic bold bases represent the mismatch on ssDNA target.

**Table S4. Nicking enzyme-assisted amplification (NEAA) probes**

| Name | Sequence (5’→3’)^a^ |  |
| --- | --- | --- |
| NEAA_Template_Target 20nt | CAC CAA ATA TCG CTA TTC G**AG AGA AGA AAG AAG GAG AAA** AAA TTG CAA TCA TCA CCA CTC C | |
| Forward primer | GCG TAT AAG TAG AGT CCA GTG GAG TGG TGA TGA TTG C | |
| Reverse primer | TCT CTA ATG TCG AGT CTA AGC ACC AAA TAT CGC TAT TC | |

a Underlined bases represent the complementary bases of the primers with the NEAA_Template_Target 20nt, while the **Bold** bases rapresent the complementary portion to the clamp portion (orange one, see Figure 1) of the Clamp-Switch module (CS_Target 20nt, see Table S2).

**Table S5. crRNA and FRET-based DNA reporter**

| Name | Sequence (5’→3’)^a^ |  |
| --- | --- | --- |
| crRNA | UAA UUU CUA CUA AGU GUA GAU *GUC UAC ACA UGG CUA AAU CU* | |
| FRET-based DNA reporter | (6-FAM)-CTC TCA **TTT TTT TTT T**AG AGA G -(BHQ 1) | |

a *Italic* bases represent the crRNA-targeting sequence. The **bold** and underlined sequences represent the loop and stem-forming portions of the FRET-based DNA reporter, respectively.

## Supplementary Figures


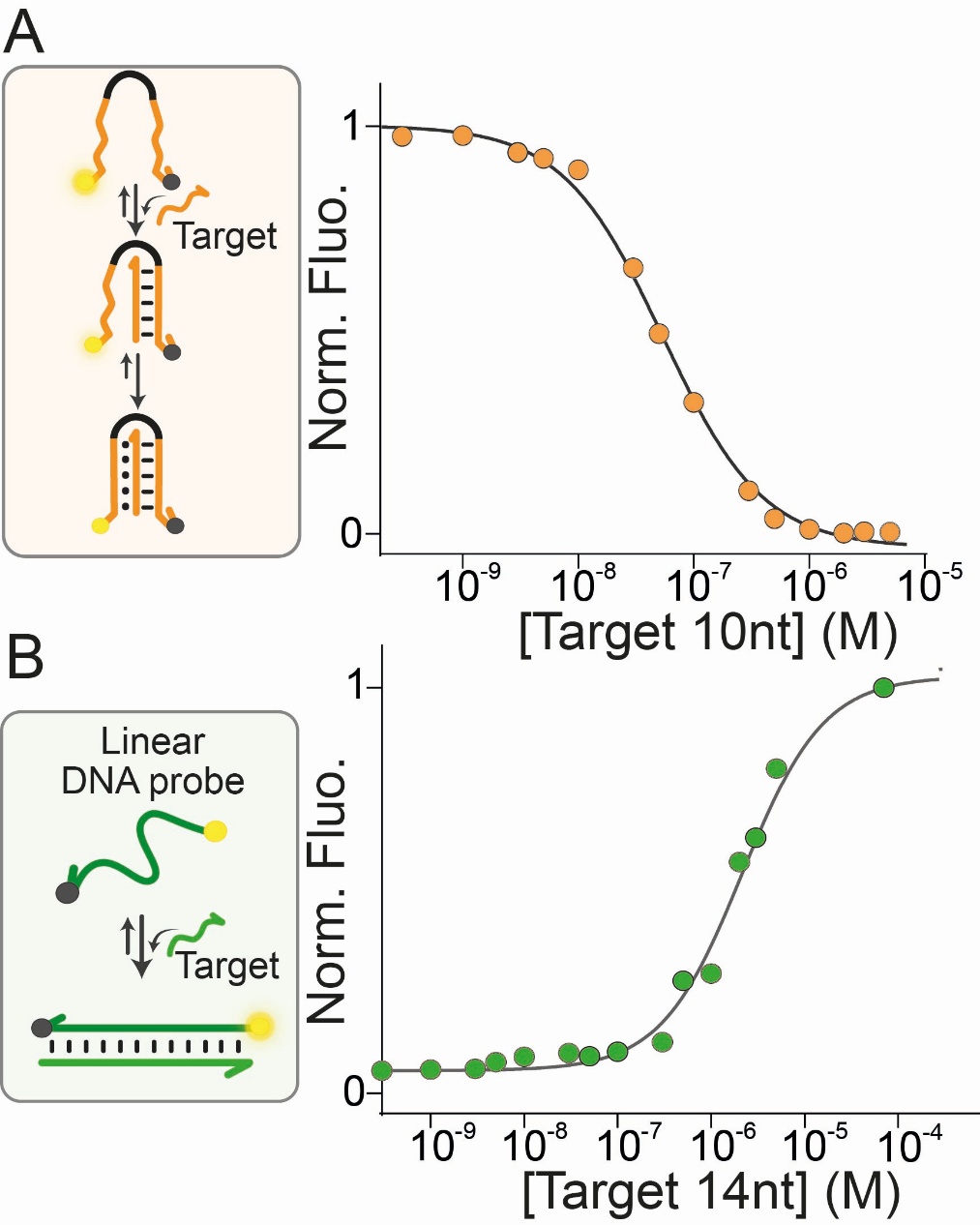


**Figure S1.** **Affinity assays using Triplex Clamp and linear DNA probes.** (**A**) Binding assay performed between the FAM/BHQ1-labelled Clamp-Switch probe (CS_FQ = 30 nM) and Target 10nt (K_D_ = 54 nM). (**B**) Fluorescence binding assay between FAM/BHQ1-labelled DNA probe (Linear DNA probe, 30 nM) and Target 14nt (K_D_ = 1.47 µM). As expected, the data indicate that the Clamp-Switch probe exhibits a higher binding affinity compared to the linear probe, as Triplex Clamp formation results in lower K_D_ even in the presence of a shorter DNA target. (1) Experiments were conducted in buffer solution (10 mM Tris-HCl, 10 mM MgCl₂, and 50 mM NaCl), pH 7.0 at T = 37°C. Fluorescence measurements were performed using a Cary Eclipse fluorimeter with excitation at 488 nm and emission at 520 nm, using 5 nm bandwidth for both excitation and emission using 50 µL quartz cuvettes.


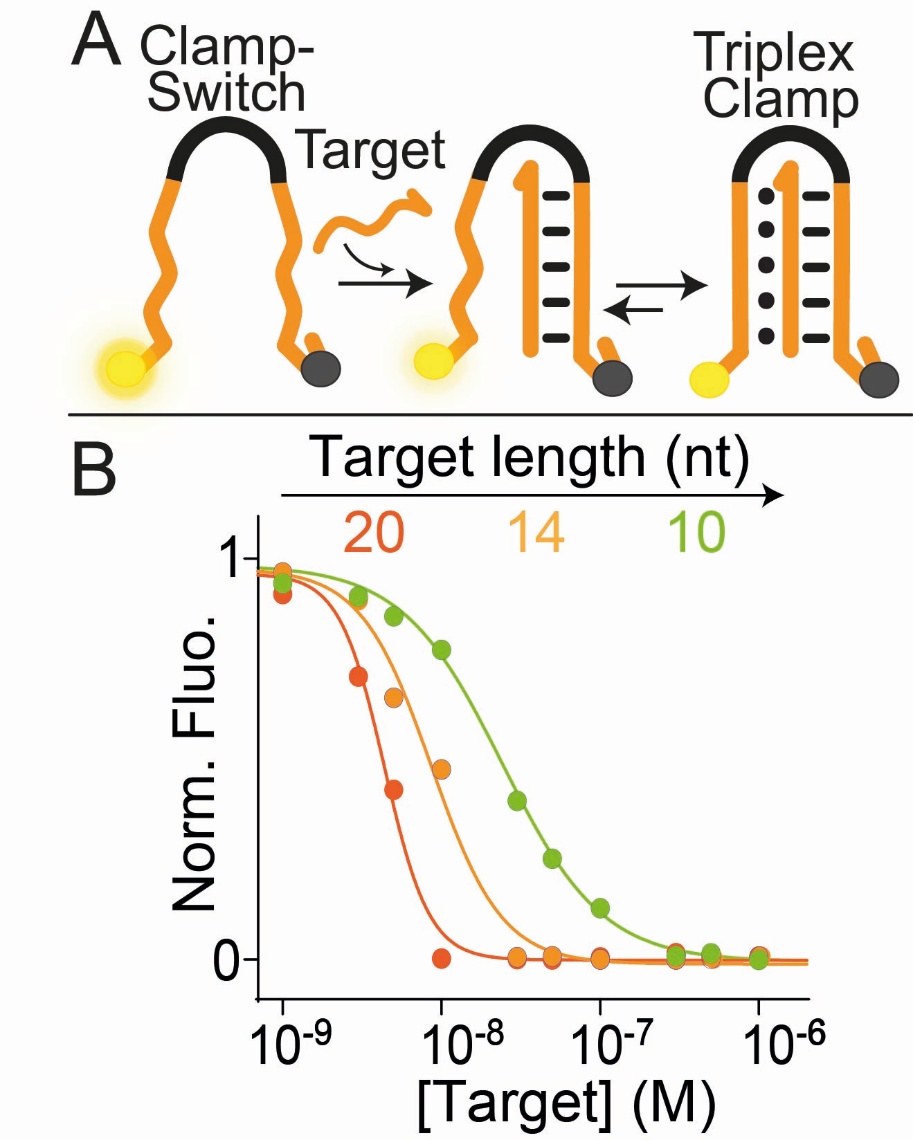


**Figure S2.** **Triplex Clamp formation in the presence of different ssDNA target lengths.** **(A)** Scheme of the triplex formation in the presence of the DNA target via clamp-like mechanism. The triplex folding results in quenching of fluorescence signal due to the close proximity of the 5’-end FAM and 3’-end BHQ1. **(B)** Fluorescence binding association curves of Target 10nt (K_1/2_ = 24 ± 2 nM), Target 14nt (K_1/2_ = 8.6 ± 1.4 nM) and Target 20nt (K_1/2_ = 4.4 ± 0.7 nM) in the presence of a fixed amount of CS_FQ (10 nM). Experiments were conducted in buffer solution (10 mM Tris-HCl, 10 mM MgCl₂, and 50 mM NaCl), pH 7.0 at T = 37°C. Fluorescence measurements were performed using a Cary Eclipse fluorimeter with excitation at 488 nm and emission at 520 nm, using 5 nm bandwidth for both excitation and emission using 50 µL quartz cuvettes.


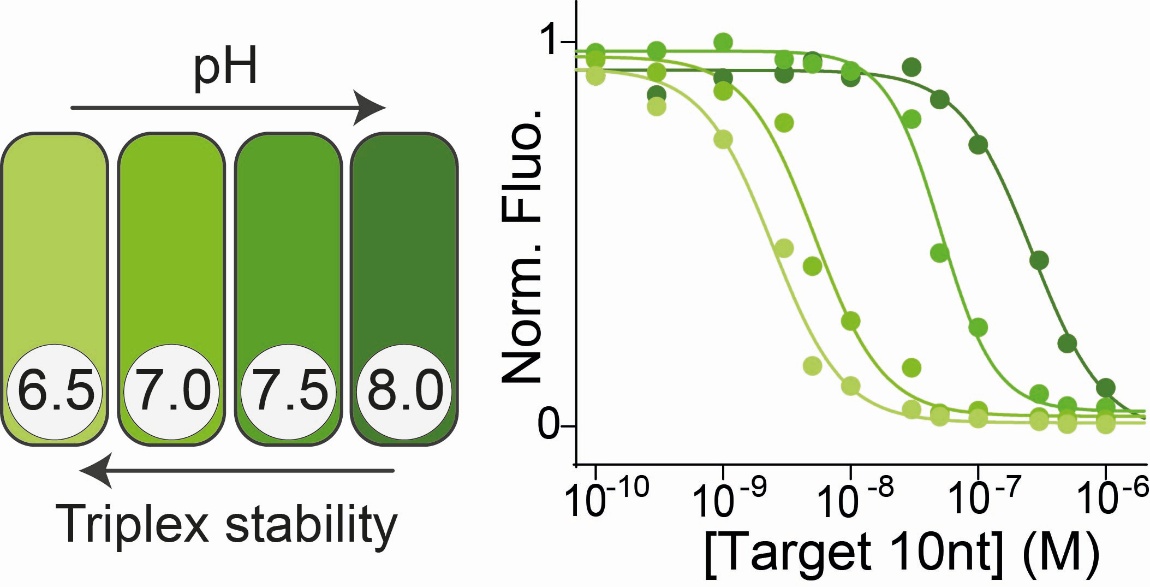


**Figure S3.** **Fluorescence binding assays of Target 10nt at different pH values confirming the pH-dependent triplex folding.** Experiments were conducted at T = 37 °C in buffer solution (10 mM Tris-HCl, 50 mM NaCl, 10 mM MgCl₂) containing a fix amount of CS_FQ (10 nM) and by adding increasing concentrations of Target 10nt. All reported values represent the results of three independent measurements.


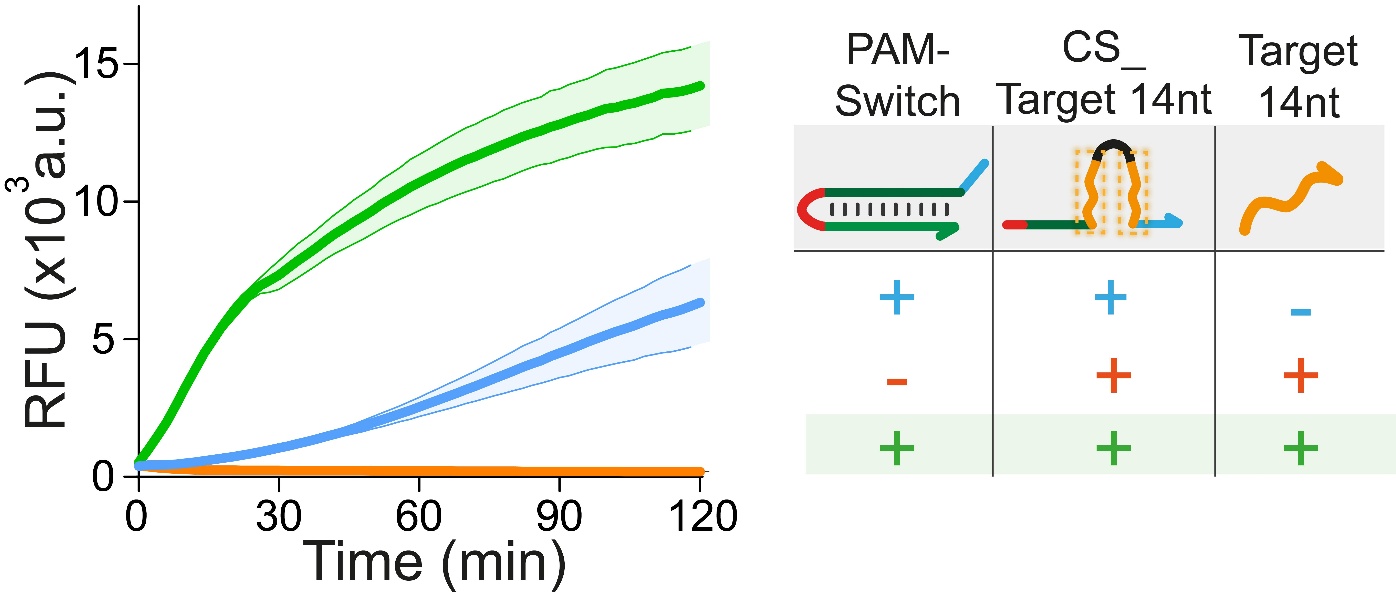


**Figure S4.** **Testing the effect of different components of the triplex-based Cas12 detection system**. Data show that Cas12a detection system is activated upon target binding (Target 14nt, 10 nM) and Triplex Clamp formation (green line). However, an upward increase of fluorescence background is observed also in the absence of target (blue line). Experiments were performed in 10 mM Tris-HCl buffer solution, 50 mM NaCl, and 10 mM MgCl₂ at pH 7.0 by adding the Cas12a reaction mixture (100 nM FRET-based DNA reporter and 20 nM RNP complex) immediatly before starting data acquisition.

##
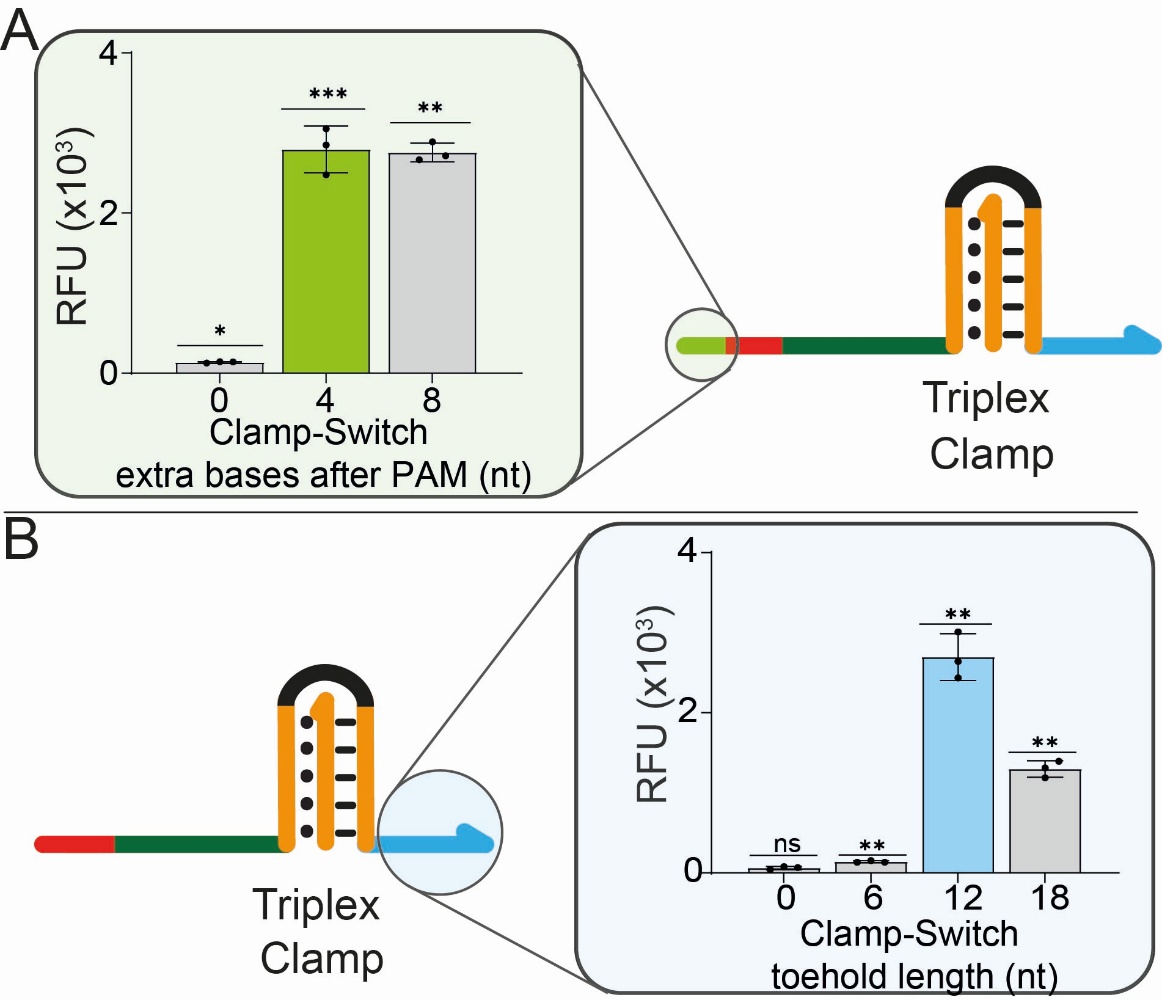


**Figure S5**. **Optimization of the Triplex Clamp module.** (**A**) Study of the 5′-terminal extensions on Triplex Clamp. Clamp-Switch variants having increasing numbers of extra nucleotides at the 5′ end (CS_Extra#0, CS_Extra#4, CS_Extra#8; see Table S2) were tested in the triplex-controlled activation of Cas12a system. (**B**) Effect of the toehold length on the detection system. Clamp-Switch variants having different toehold regions (CS_Toehold_0, CS_Toehold_6, CS_Target 14nt, CS_Toehold_18; see Table S2) were tested using corresponding PAM-Switch variants (PAM-Switch_Toehold0, PAM-Switch_Toehold6, PAM-Switch, PAM-Switch_Toehold18; see Table S1). Experiments were performed at 37 °C in a buffer (10 mM Tris-HCl, 50 mM NaCl, and 10 mM MgCl₂ at pH 7.0), containing PAM-Switch (0.5 nM), Clamp-Switch variants (20 nM), and Cas12a reaction mix (100 nM FRET-based DNA reporter and 20 nM RNP complex), following the addition of 10 nM Target 14nt. Data represents RFU mean values (t= 15 min) ± SD from three independent replicates. Statistical significance versus the respective no-target control was determined by unpaired t-test with Welch’s correction: P < 0.0001 (****), P < 0.001 (***), P < 0.01 (**), and P ≤ 0.05 (*).

##
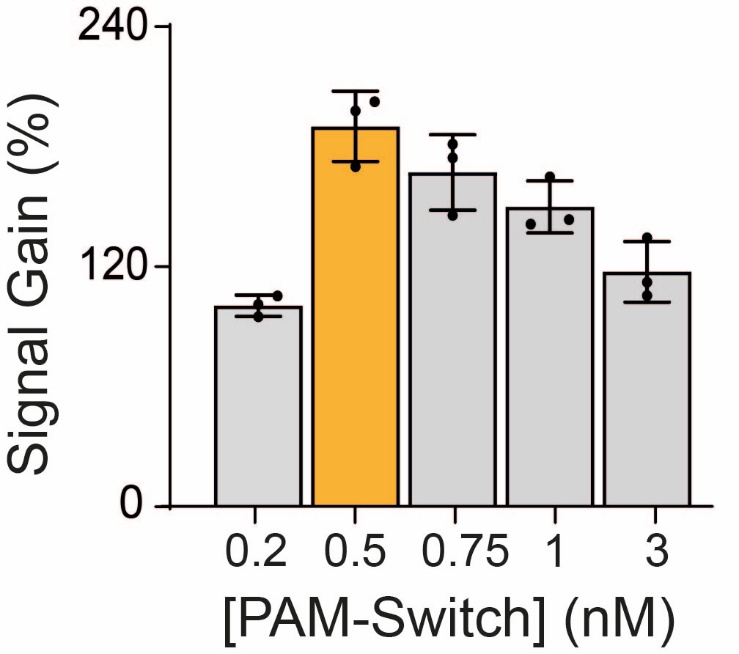


**Figure S6.** **Optimization of PAM-Switch concentration.** The experiments were performed at 37 °C by testing different concentrations of PAM-Switch in the standard buffer solution containing CS_Target 14nt (20 nM) and Target 14nt (10 nM) by adding the Cas12a reaction mixture (100 nM FRET-based DNA reporter and 20 nM RNP complex). The reported Signal Gain (%) was calculated after 15 minutes from the start of the reaction. Error bars represent the standard deviation from three independent replicates.


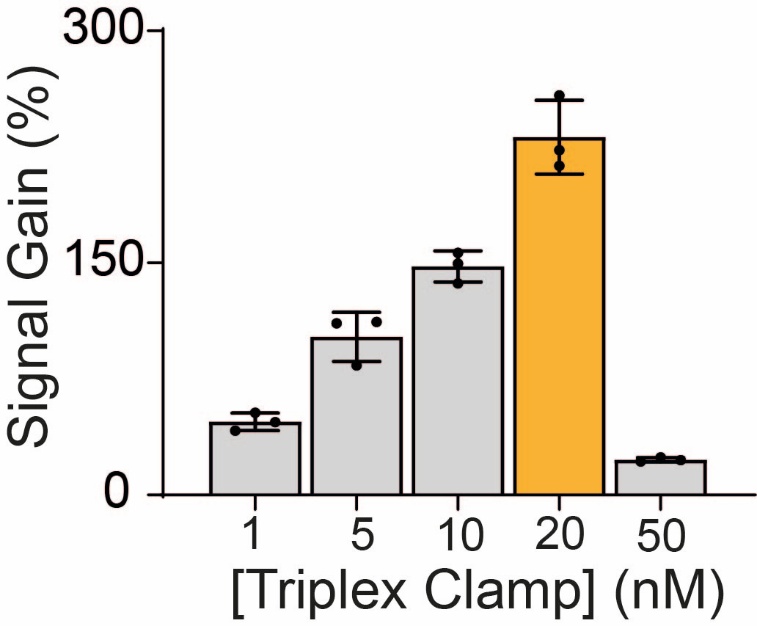


**Figure S7.** **Optimization of Clamp-Switch concentration.** The experiments were performed at 37 °C by testing different concentrations of Triplex Clamp in the standard buffer solution containing PAM-Switch (0.5 nM) and Target 14nt (10 nM) by adding the Cas12a reaction mixture (100 nM FRET-based DNA reporter and 20 nM RNP complex). The reported Signal Gain (%) was calculated after 15 minutes from the start of the reaction. Error bars represent the standard deviation from three independent replicates.


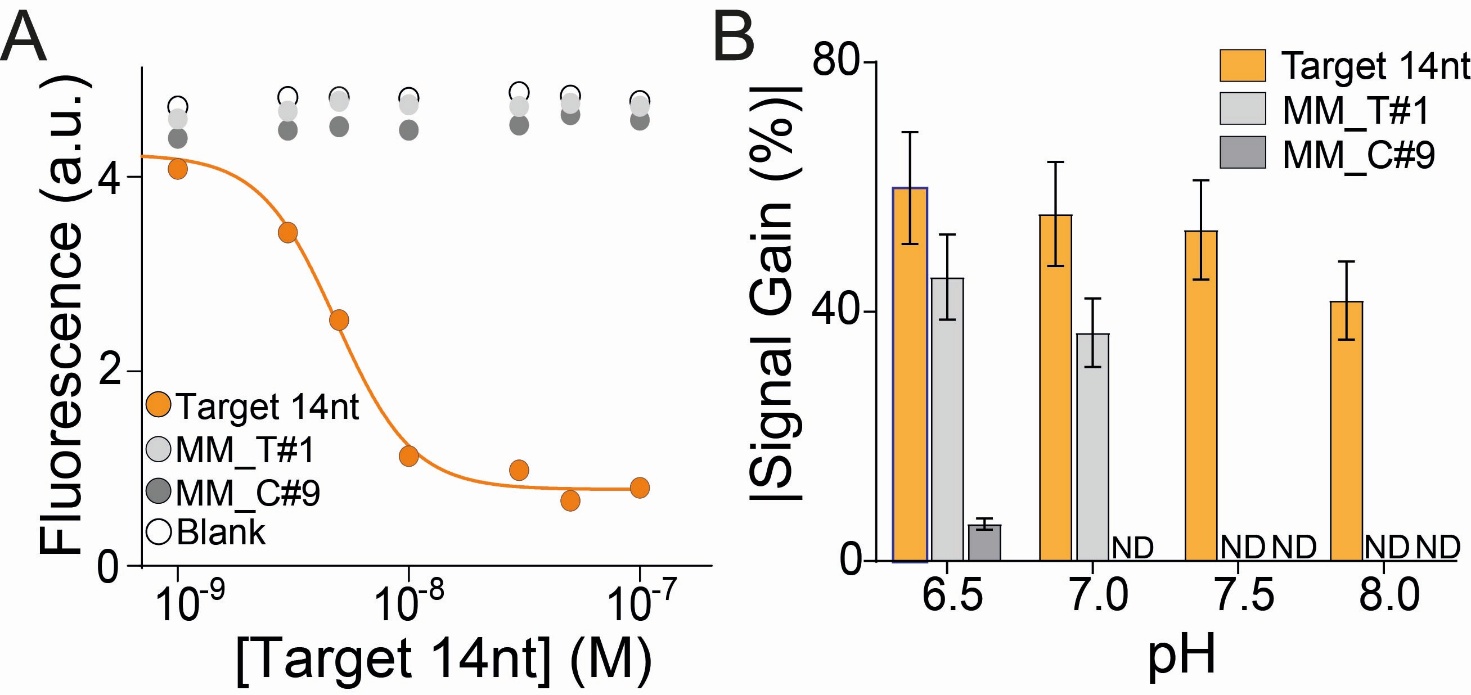


**Figure S8.** **Specificity tests using the FRET-labelled Clamp Switch probe.** (**A**) Binding curves showing the formation of the Triplex Clamp as a function of DNA target concentration obtained using Target 14nt or two single-mismatch Target 14nt variants (MM_T#1 and MM_C#9) in the presence of a fixed amount of the Clamp-Switch (CS_FQ). (**B**) The graph illustrates the pH-dependent change in the specificity of the Clamp-Switch. Experiments were conducted at 37 °C in a buffer (10 mM Tris-HCl, 50 mM NaCl, and 10 mM MgCl₂), with 10 nM CS_FQ and 5 nM of either the fully matched target (Target 14nt) or single-mismatch variants (MM_T#1 and MM_C#9). Increasing the pH of the solution enhances the discrimination between matched and mismatched sequences. Signal Gain (%) was calculated after 15 min of Cas12 trans cleavage. Data represent mean ± SD from three independent replicates; ND (non-detectable) indicates values below the blank signal, considered not detectable in this assay. Measurements were performed in a volume of 50 µL using quartz cuvettes in a Cary Eclipse fluorimeter.


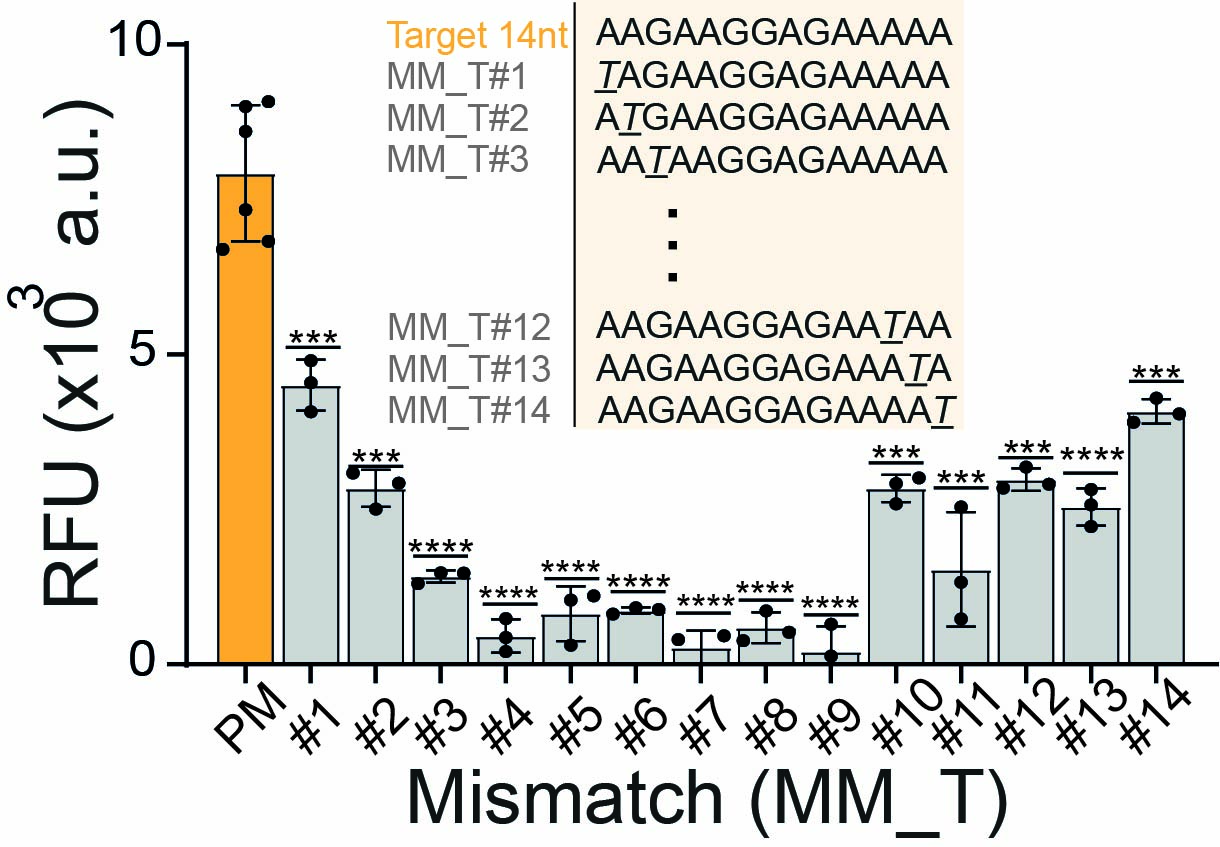


**Figure S9. Bar graphs comparing fluorescence outputs generated by fully matched (PM, Target 14nt) and single-nucleotide mismatch (MM) targets.** Reactions were performed at 37 °C in a buffer (10 mM Tris-HCl, 50 mM NaCl, and 10 mM MgCl₂ at pH 7.5), using a fixed concentration of PAM-Switch (0.5 nM), Clamp-Switch CS_14 (20 nM), and either fully matched (Target 14nt) or single-mismatch (MM_T) targets at 5 nM. Following a 15-minute pre-incubation, the Cas12a reaction mix (100 nM FRET-based DNA reporter, 20 nM RNP complex) was added, and fluorescence was collected after 15 minutes of reaction. Data represent a mean value ± standard deviation (SD), n = 3 independent replicates. Asterisks indicate statistical significance relative to the PM (perfect match) sequence, calculated using an unpaired t-test with Welch's correction (n = 3): P < 0.0001 (****), P < 0.001 (***), P < 0.01 (**), and P ≤ 0.05 (*).


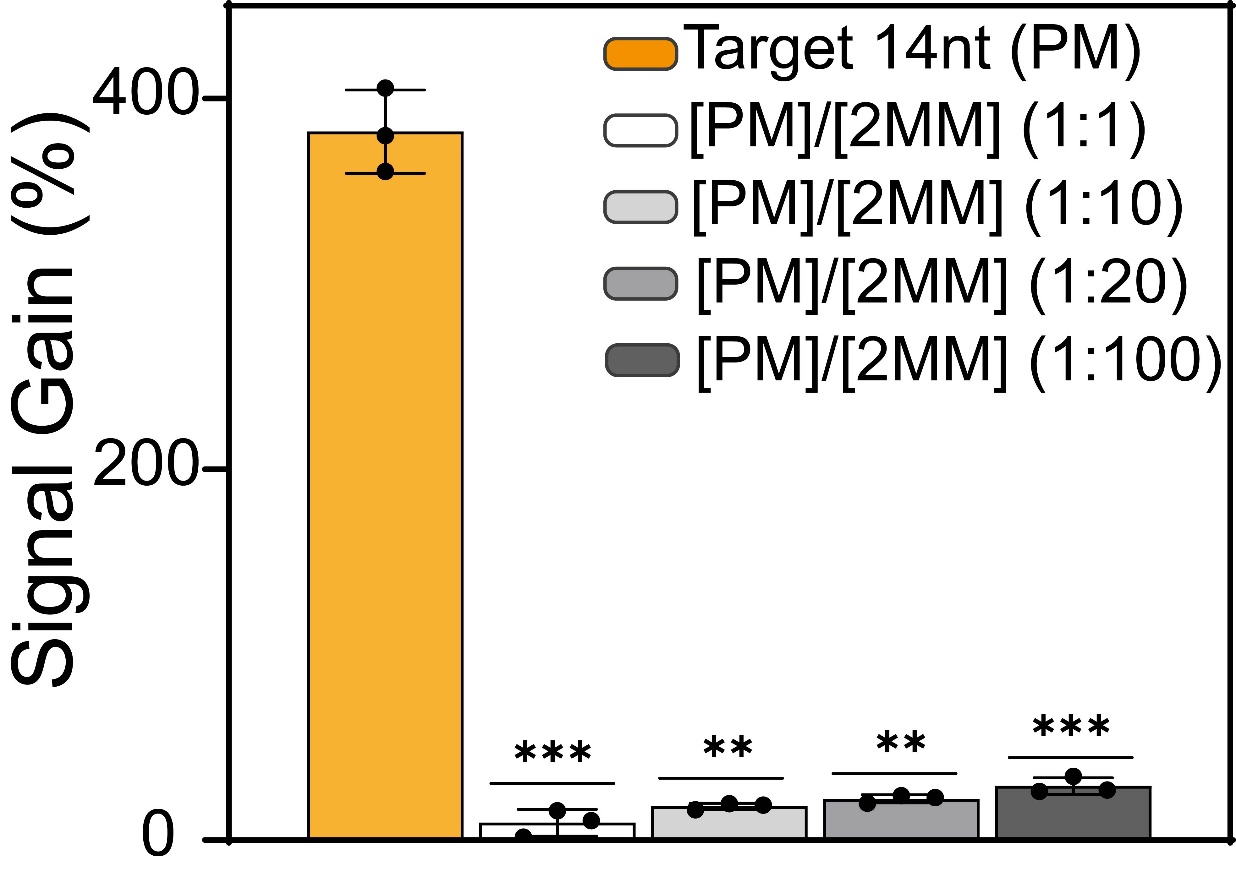


**Figure S10. Bar graph showing that a molar excess of double-nucleotide mismatch DNA target (2MM, Target 14nt_2MM) does not compromise the specificity**. Signal gain generated by fully matched (PM, Target 14nt) and double-nucleotide mismatch (2MM, Target 14nt_2MM) targets in molar excess. Reactions were carried out at T = 37 °C in a buffer (10 mM Tris-HCl, 50 mM NaCl, and 10 mM MgCl₂ at pH 7.5), using fixed concentrations of PAM-Switch (0.5 nM) and Clamp-Switch CS_14 (20 nM), along with either fully matched targets (Target 14nt, 10 nM) or double-mismatch (2MM) targets at different concentrations (10 nM, 100 nM, 200 nM, and 1 µM). After a 15-minute pre-incubation, the Cas12a reaction mix (100 nM FRET-based DNA reporter, 20 nM RNP complex) was added, and fluorescence was measured after 15 minutes. Data are presented as mean ± SD from three independent replicates (n = 3). Statistical significance relative to the perfectly matched (PM) sequence was determined using an unpaired t-test with Welch’s correction: P < 0.0001 (), P < 0.001 (*), P < 0.01 (), and P ≤ 0.05 (*).


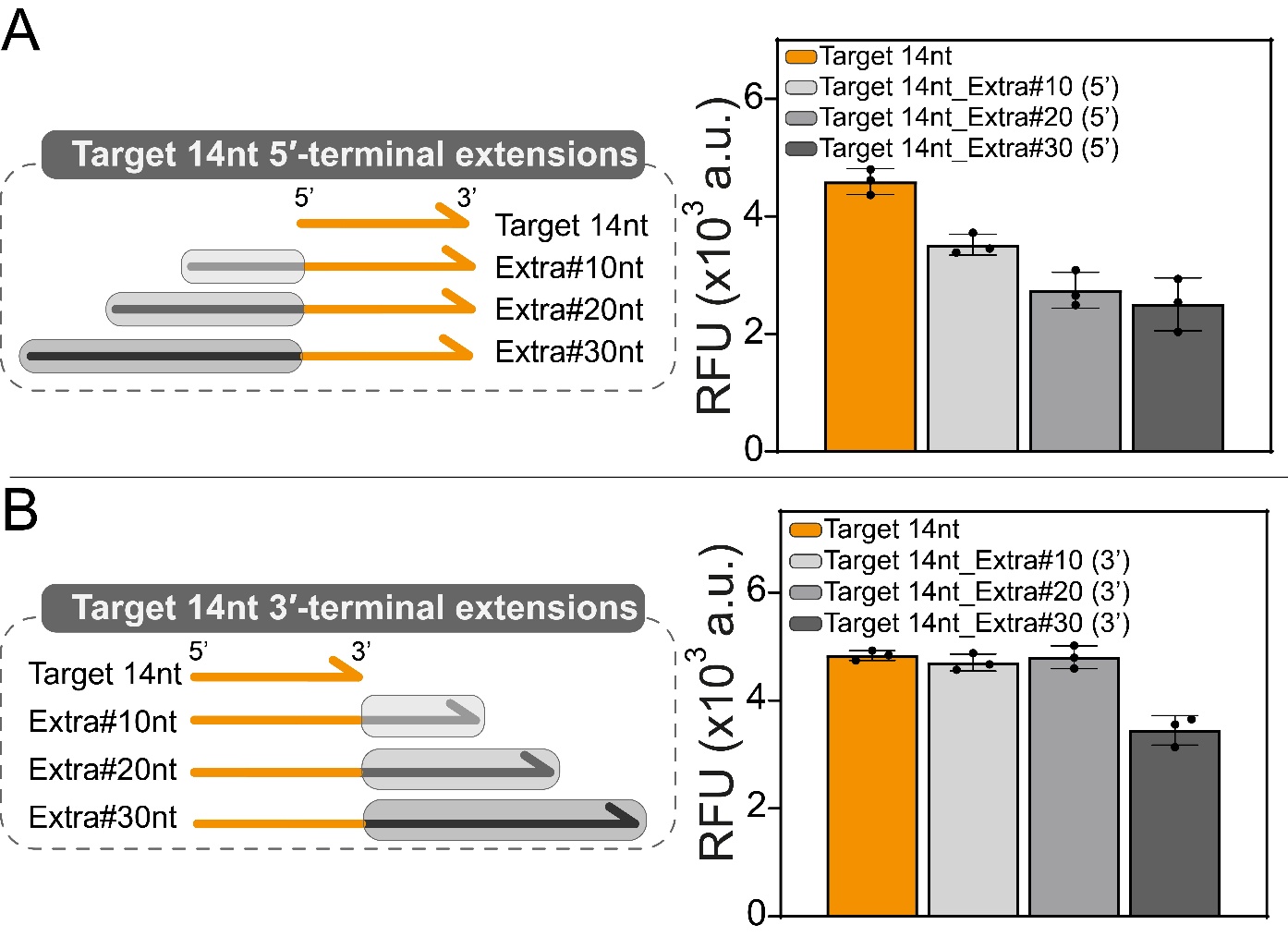


**Figure S11.** Impact of 5′- and 3′-terminal overhangs on the Triplex-controlled CRISPR–Cas12a system. (**A**) 5′-terminal overhangs affects the overall signal transduction of the Cas12a-based detection system in a length-dependent way. However, the detection system can detect the specific DNA target even in the presence of overhangs. (**B**) Adding 3′- overhangs does not significantly affect the assay until a threshold of extra 30 nt. Experiments were performed at T = 37 °C in buffer solution (10 mM Tris–HCl, 50 mM NaCl, and 10 mM MgCl₂), using 20 nM CS_Target 14nt and 0.5 nM PAM-Switch in the presence of 10 nM of either the fully matched DNA target (Target_14nt) or targets carrying 5′- or 3’- overhangs. Data represent mean value ± standard deviation (SD) from three independent experiments (n = 3).


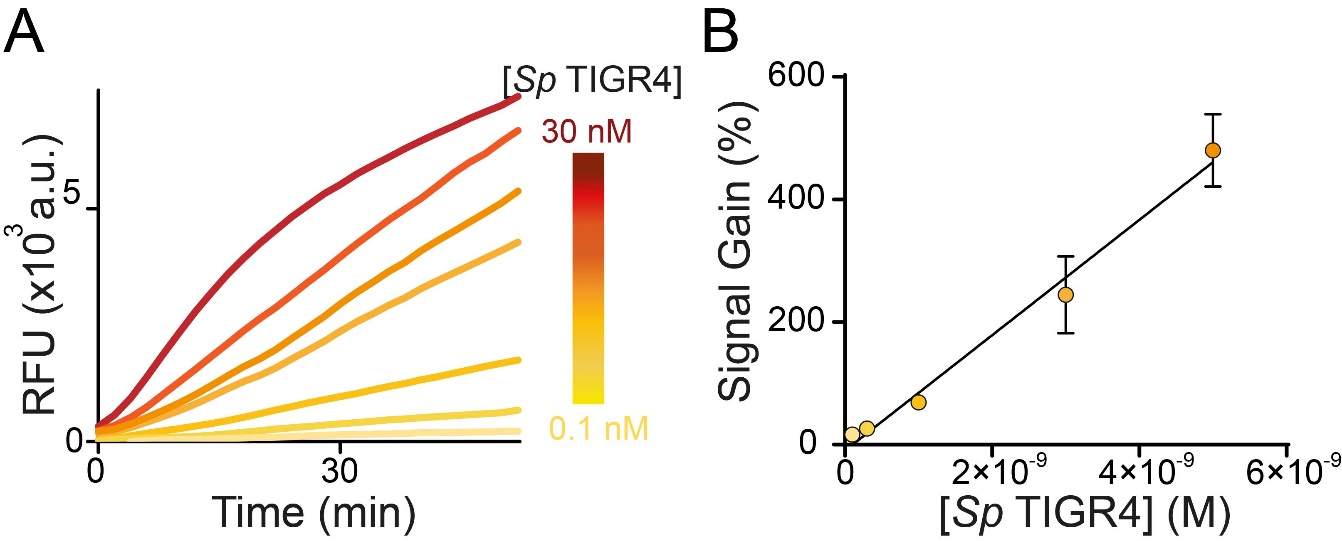


**Figure S12.** (A) Time-dependent fluorescence kinetics showing the detection of Sp TIGR4 (0.1, 0.3, 1, 3, 5, 10, and 30 nM). (B) Linear dynamic range of Sp TIGR4 detection comprised between 0.1 and 5 nM (R² = 0.9660, n = 3; LOD = 676 pM), as determined from the plot of fluorescence signal change (gain) versus target concentration. Signal gain (%) was calculated after 30 min of cleavage activity and represents the relative fluorescence change associated with collateral cleavage upon addition of Sp TIGR4.


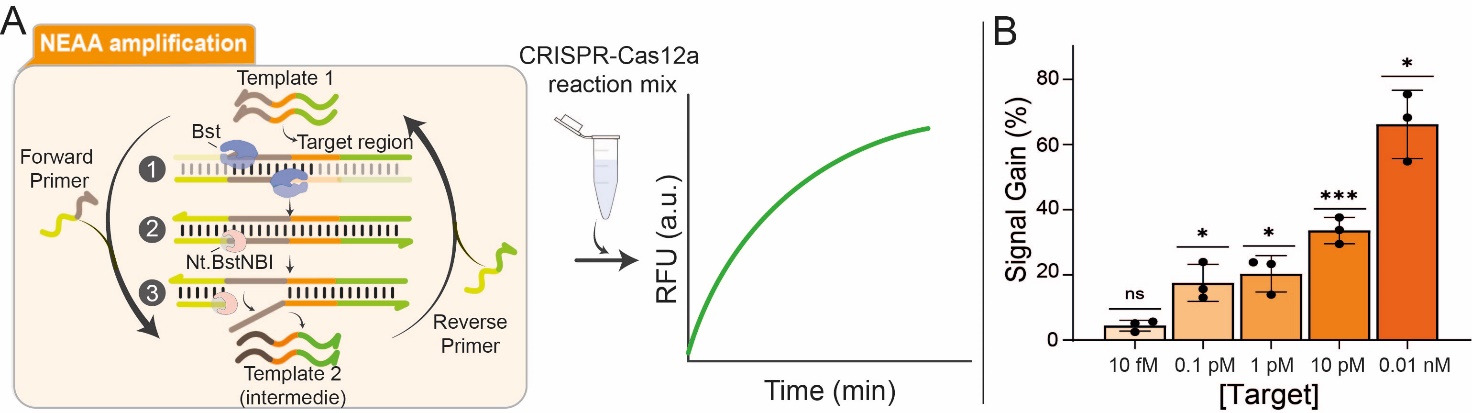


**Figure S13. Triplex-based Cas12a detection platform integrated with nicking enzyme-assisted amplification (NEAA).** (**A**) Schematic representation of the NEAA strategy coupled with the triplex-based Cas12a detection system. (**B**) Two-pot detection of NEAA-amplified DNA template (NEAA_Template_Target 20nt, see Table S4) at concentrations ranging from 10 fM to 0.01 nM. For experimental details check the Materials and Methods. Signal Gain (%) was calculated after 15 min. Data are presented as mean ± SD (n = 3). Asterisks indicate statistical significance relative to the background signal, calculated using an unpaired t-test with Welch's correction (n = 3): P < 0.0001 (****), P < 0.001 (***), P < 0.01 (**), and P ≤ 0.05 (*).


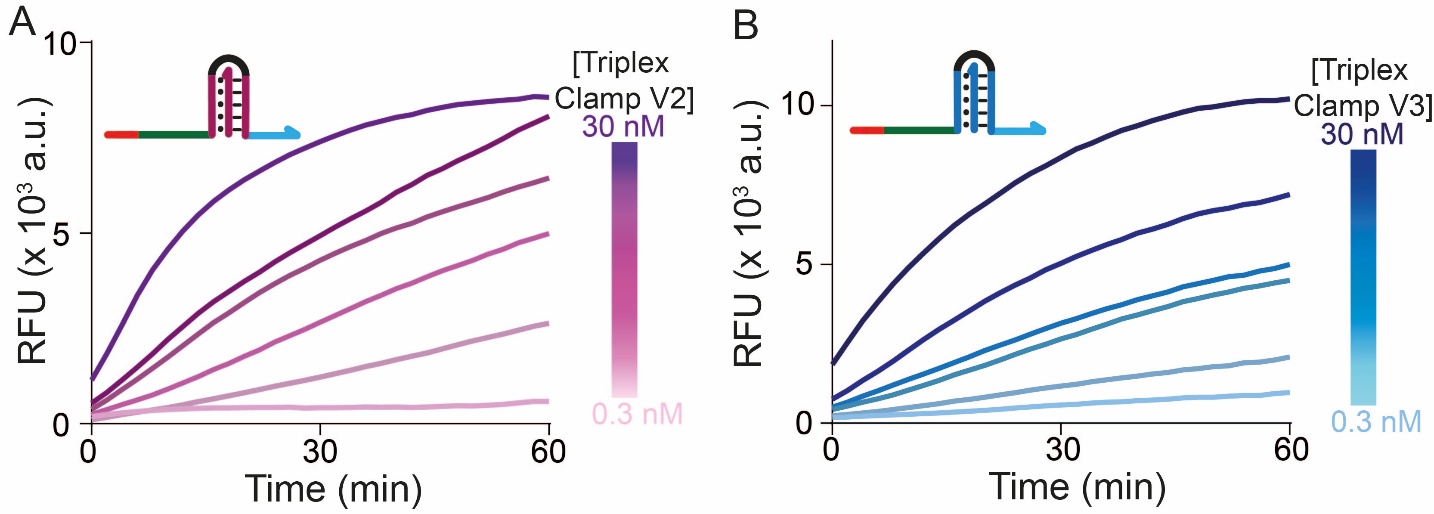


**Figure S14.** Fluorescent kinetic traces of Cas12a trans-cleavage activity using different Clamp-Switch variants (CS_Target 14nt_V2 (**A**) and CS_ Target 14nt _V3 (**B**)), in the presence of their corresponding Target 14nt targets (Target 14nt _V2 (**A**) and Target 14nt _V3 (**B**)). Experiments were conducted at 37 °C in a buffer solution (10 mM Tris-HCl, 50 mM NaCl, 10 mM MgCl₂, pH 7.0). Various concentrations of ssDNA targets (Target 14nt _V2 and Target 14nt _V3; 0.3 nM, 1 nM, 3 nM, 5 nM, 10 nM, 30 nM) were added to a reaction mix containing PAM-Switch (0.5 nM) and the specific Clamp-Switch variants (20 nM).

***References***

1. Idili A, Plaxco KW, Vallée-Bélisle A, Ricci F. Thermodynamic basis for engineering high-affinity, high-specificity binding-induced DNA clamp nanoswitches. ACS Nano. 2013 Dec 23;7(12):10863–9. doi: 10.1021/nn404416v.
2. Bagheri N, Chamorro A, Idili A, Porchetta A. PAM‐Engineered Toehold Switches as Input‐Responsive Activators of CRISPR‐Cas12a for Sensing Applications. Angewandte Chemie. 2024 Apr 22;136(17):e202319677. 10.1002/anie.202319677
